# Direct observation of chaotic resonances in optical microcavities

**DOI:** 10.1038/s41377-021-00578-7

**Published:** 2021-06-30

**Authors:** Shuai Wang, Shuai Liu, Yilin Liu, Shumin Xiao, Zi Wang, Yubin Fan, Jiecai Han, Li Ge, Qinghai Song

**Affiliations:** 1 Ministry of Industry and Information Technology Key Lab of Micro-Nano Optoelectronic Information System, Harbin Institute of Technology (Shenzhen), Shenzhen, 518055 China; 2 National Key Laboratory of Science and Technology on Advanced Composites in Special Environments, Harbin Institute of Technology, Harbin, 150080 China; 3 Collaborative Innovation Center of Extreme Optics, Shanxi University, Taiyuan, 030006 China; 4 Department of Physics and Astronomy, College of Staten Island, CUNY, Staten Island, NY 10314 USA; 5 The Graduate Center, CUNY, New York, NY 10016 USA

**Keywords:** Microresonators, Micro-optics

## Abstract

Optical microcavities play a significant role in the study of classical and quantum chaos. To date, most experimental explorations of their internal wave dynamics have focused on the properties of their inputs and outputs, without directly interrogating the dynamics and the associated mode patterns inside. As a result, this key information is rarely retrieved with certainty, which significantly restricts the verification and understanding of the actual chaotic motion. Here we demonstrate a simple and robust approach to directly and rapidly map the internal mode patterns in chaotic microcavities. By introducing a local index perturbation through a pump laser, we report a spectral response of optical microcavities that is proportional to the internal field distribution. With this technique, chaotic modes with staggered mode spacings can be distinguished. Consequently, a complete chaos assisted tunneling (CAT) and its time-reversed process are experimentally verified in the optical domain with unprecedented certainty.

## Introduction

The detailed understanding of mixed-phase space with both regular and chaotic motions is essential for a wide range of physical systems, ranging from atomic and molecular physics to mesoscopic science and even astrophysics^1–3^. Among the cornucopia of quantum chaotic phenomena in mixed-phase space, the chaos assisted tunneling (CAT) between distinct regular phase space regions separated by a chaotic sea is particularly interesting and has been intensively studied in molecular dynamics, cold atom assemblies, and whispering gallery modes (WGM) microcavities^4–7^. Among these systems, WGM microcavities have a series of advantages to study CAT, including easy control of internal quantum or classical dynamics and multiple manifestations of CAT beyond the regular level splitting^7–19^. In past decades, the rapid progress in WGM microcavities^20–22^ has not only enriched fundamental physics such as CAT, resonance assisted tunneling^23^, and turnstile transport^24^, but also enabled many practical applications from broadband coupling^16,25,26^ to integrated laser sources^27–29^, sensors^30,31^ and all-optical switches^32^.

Despite these achievements, the conventional study of mixed-phase space in optical WGM microcavities focuses on its ancillary signatures in the inputs and the outputs, without directly interrogating the dynamics and the associated mode patterns inside. These techniques include, for example, comparing the experimentally observed free spectral range (FSR) and far-field radiation patterns with theoretical predictions^11,12,18,19,33–36^. However, such comparisons often lead to educated conjectures instead of conclusive affirmation, especially when multiple mode groups have similar FSRs and far-field patterns^37,38^. The association of internal mode patterns with experimentally observed resonances is hence crucial to provide a clearer picture of quantum chaos in optical microcavities.

Inspired by a technique developed in the microwave domain^9,39,40^, here we propose and demonstrate a simple, robust, and contactless mechanism to rapidly map the field patterns in silicon microdisks. Compared with other attempts^10,41,42^ at visualizing optical modes inside a cavity, our method allows the observation of modes with drastically different dynamics, covering phase space structures such as unbroken Kolmogorov–Arnold–Moser (KAM) curves, stable periodic motions, and the chaotic sea. Using this technique, we report the observation of the complete process of CAT and its time-reversed counterpart with unprecedented assurance in the optical domain.

## Results

### Field mapping principle

**Fig. 1:**
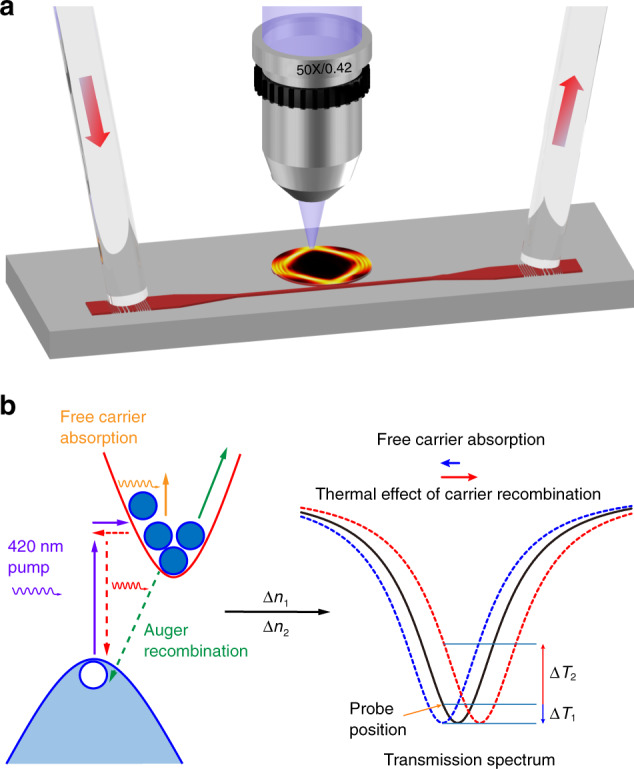
The working principle and schematic of the mapping setup. **a** After passing through the single-mode fiber and grating coupler, the probe light is coupled into the silicon microdisk via evanescent coupling from the Si waveguide. **b** When a 420 nm nanosecond laser is focused onto the top surface of the microdisk, local refractive index changes (∆*n*_1_ and ∆*n*_2_ resulted from FCA and thermal effect, respectively) induce the shifting of the resonance frequencies. For a fixed probe wavelength, the shifts of resonances lead to two opposite changes of transmittance ∆*T*_1_(*x, y*) and ∆*T*_2_(*x, y*) that can be measured at the output port

Figure 1 shows the schematic of the working principle. The setup utilizes the conventional measurement scheme for on-chip integrated silicon microdisks (see Supplementary Section 1), with the crucial addition of a nanosecond laser at 420 nm (6 ns pulse duration, 10 Hz repetition rate) normally focused on the microdisk through a 50× objective lens (NA = 0.42). The probe laser in the single-mode fiber is tuned to the shorter wavelength side of each resonance. The above-bandgap optical excitation induces free-carrier absorption (FCA) as well as thermal heating within the silicon, both of which lead to induced local changes of the refractive index by ∆*n*_1_ and ∆*n*_2_^41,43^, respectively. For the range of pump laser in our experiments, we estimate that ∆*n* is on the order of 0.01 or smaller. The mode patterns in our system are only minutely perturbed and keep their geometric character, e.g., as a WGM or localized on a periodic orbit (see Supplementary Section 2). Correspondingly, the resonant wavelengths shift, following the equation

1 $${\Delta}\lambda (x_0,\;y_0) = \lambda _0{\int} {\frac{{{\Delta}n\left( {x,\;y;\;x0,\;y0} \right)}}{n}} \psi _0^2\left( {x,\;y} \right)dxdy$$

in the absence of strong mode mixing^44^. Here *λ*_0_ is the unperturbed resonant wavelength, *n* is the unperturbed cavity refractive index, a constant for our optical microcavities. Δ*n*(*x*, *y*; *x*_0_, *y*_0_) is its change due to the pump beam centered at (*x*_0_, *y*_0_), and $\psi _0\left( {x,\;y} \right)$is the corresponding out-of-plane *H* field normalized by ${\int} {\psi _0^2\left( {x,\;y} \right)} \;dxdy = 1.$ For a fixed value of Δ*n*, Eq. (1) shows clearly that the wavelength shift is proportional to the field distribution (i.e., $\psi _0^2\left( {x,\;y} \right)$) at the pump spot. As a result, by performing a two-dimensional scan on the surface of the microdisk with a fixed pump density, the field pattern inside can be read out from the corresponding wavelength shift in the transmission spectrum. However, instead of measuring this wavelength shift directly, an easier approach is to measure the change of the transmission at a fixed wavelength, set by the probe laser close to each unperturbed resonance (See the discussion on the influence of the probe wavelength in Supplementary Section 2). Here we note that ∆*n*_1_ and ∆*n*_2_ due to FCA and thermal heating have opposite signs, and so are the resulting changes of the transmission (denoted by ∆*T*_1_(*x*_0_, *y*_0_) and ∆*T*_2_(*x*_0_, *y*_0_)) in Fig. 1b. Since the former is on the order of noise in our experiment, only the latter is extracted to represent the field distribution (see detailed discussion in Supplementary Section 3).

### Verification in circular microcavity

**Fig. 2:**
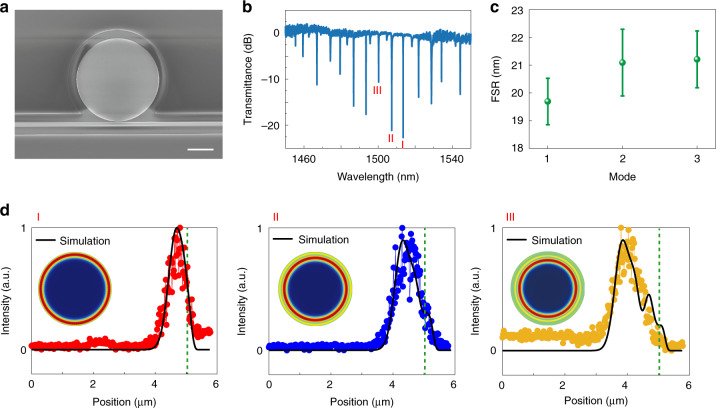
Validation of the proposed method in a circular microdisk. **a**. Top-view SEM image of a silicon microdisk. The radius of the microdisk is 5 µm, the widths of the bus waveguide and the gap are 458 nm and 80 nm, respectively. The scale bar is 3 µm. **b**. Experimentally recorded transmission spectrum along the bus waveguide. **c**. Average FSR of three sets of resonances in the silicon microdisk. **d**. Experimentally recorded field distributions of resonances I, II, III in Fig. 2b. Here only the intensity along the radius is considered due to the rotational symmetry. Scan along the horizontal axis, starting from the center of the cavity to the right edge. The numerically calculated field distributions after taking the 600 nm laser spot size into account are shown as solid lines for a direct comparison. The insets are the corresponding numerically calculated traveling wave field patterns

To validate the proposed measurement scheme, we apply it to a circular silicon microdisk fabricated on a 220 nm silicon on insulator (SOI) wafer with a combined process of electron beam lithography and reactive ion etching (see “Methods”). Figure 2a shows the top-view scanning electron microscope (SEM) image of the silicon microdisk. It is a circular disk with *R* = 5 µm coupled to a bus waveguide. The transmission spectrum is recorded at the throughput of the bus waveguide and plotted in Fig. 2b. Different from an active microlaser with an embedded gain medium, here a large number of resonances with different quality (*Q*) factors can be resolved in this passive microdisk. Taking the modes marked by I, II, III in Fig. 2b as an example, the FWHMs are 0.110 nm, 0.305 nm, 0.145 nm and the corresponding *Q* factors are 13759, 4950, and 10347, respectively (see details in Supplementary Section 8). It was commonly hypothesized that the field distribution of a particular WGM can be obtained by analyzing its FSR and *Q* factor. However, such an approach is at most an educated conjecture in nanofabricated systems, where the shape of the microdisk deviates minutely from the designed one. For example, using a simple estimation of the FSR given by $\lambda ^2/2\pi n_{group}R$, the most prominent resonances in Fig. 2b can be categorized into three groups. As shown in Fig. 2c, their respective FSRs are very close and hence cannot be used reliably to deduce the spatial features of the corresponding WGMs; their *Q* factors do not display a systematic difference, either.

In contrast, our method based on the local index variation gives convincing and clearly distinct results. For mode I to III, one from each group mentioned earlier. We are able to map their field distributions along the cavity radius with the setup in Fig. 1a. The pump density is fixed at 1 nJ (µm^2^)^−1^, and the probe laser is tuned to 1513.489 nm, 1507.331 nm, and 1500.258 nm for three modes, respectively. The experimental results are summarized using connected dots of different colors in Fig. 2d, from which it is clear that modes I–III are most intense at 0.33 µm, 0.8 µm, and 1.14 µm away from the disk boundary (green dashed lines), respectively. In the meanwhile, we also observe the increase of the full width at half maximum (FWHM) of the field distribution from mode I to III. These features are consistent with the simulated WGMs after taking the focal spot size into account (solid lines in Fig. 2d), indicating that the three aforementioned groups of resonances have radial quantum numbers of *j =* 1, 2, 3, respectively, which cannot be retrieved indisputably using conventional methods.

In our experiment, the laser spot size is around 600 nm (see Supplementary Section 6), which depends on the pump wavelength and the NA of objective lens. Considering the diffusion of thermal heating effect, the final resolution is determined by the laser spot size and thermal diffusivity in silicon (see details in Supplementary Section 6). While the transverse structures of propagation mode can be resolved, the laser spot size can still be improved to 200–300 nm by employing shorter pump wavelength, larger NA objective lens, and high-speed photodetector. The scanning speed can also be further increased with the assistance of Galvo scanning mirror (see details in Supplementary Section 6).

### Mapping different field patterns in one deformed microcavity

**Fig. 3:**
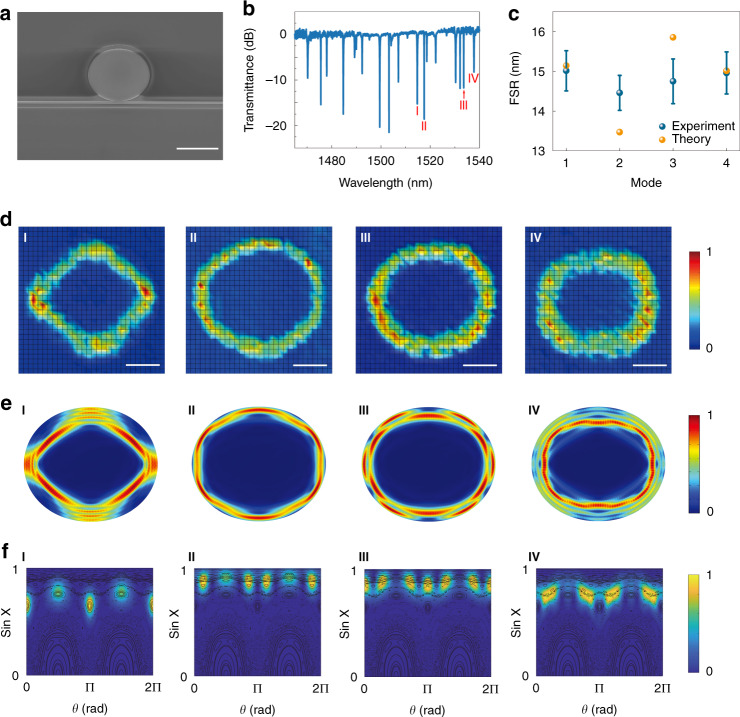
Mapping resonances in the deformed silicon microdisk. **a** Top-view SEM image of the quadrupolar microdisk. Here *R* = 7 µm and *ε* = 0.08. The width of bus waveguide and the gap size are 462 nm and 80 nm, respectively. The scale bar is 10 µm. **b** Experimentally recorded transmission spectrum along the bus waveguide. **c** Mode spacings of four groups of high *Q* resonances. Orange dots show their analytical values in an ideal quadruple. **d** Experimentally mapped field distributions of mode I-IV. The scale bars are 4 µm. **e** and **f** the corresponding numerically calculated traveling wave mode patterns and their Husimi maps

Assured by these definitive findings, we proceed to consider the quadruple cavity and its CAT process. The cavity shape is defined by *ρ*(*θ*) = *R* (1 + *ε* cos2*θ*) in polar coordinates, and the classical ray dynamics become increasingly chaotic as the deformation parameter *ε* becomes larger^7^. An example of the Poincaré surface of section (PSOS) at *ε =* 0.08 is given in the in the Supplementary Section 4, showing a mixture of regular and chaotic regions. It consists of unbroken KAM curves close to sin *χ* = 1, representing the quasi WGMs. At intermediate sin *χ* ~ [0.4, 0.9], there are islands corresponding to stable periodic motions, which are surrounded by the chaotic regions in the PSOS and above the critical angle *χ*_*c*_ = sin^−1^ (1/*n*) in silicon. As such, this quadruple microdisk, fabricated with the same process as above (see Fig. 3a), provides an ideal platform for the study of CAT and other chaotic motions.

Due to the complex phase space structures, multiple resonances with similar high *Q* factors exist that are confined by total internal reflection along stable periodic orbits or by wave localization in the chaotic sea, leading to a more complicated transmission spectrum (see Fig. 3b) compared with the circular disk. Nevertheless, four groups of modes can be separated by estimating the FSRs of periodic orbits again. As depicted in Fig. 3c, their FSRs are even closer than the circular disks and strongly overlap, and it is extremely challenging to identify the modes confined on stable islands with certainty. Even if the far-field angular distributions of these resonances are measured with an infrared camera, to associate them with a particular CAT process in the quadruple microdisk is still merely an assumption. This is because that the regular modes within stable islands usually have similar far-field emissions as their chaotic counterparts.

The above difficulty can be solved by mapping the field patterns with the setup introduced in Fig. 1a. Modes I–IV, one from each of the four groups, are selected and experimentally studied. Here the pump laser scans 33 steps in both the horizontal and vertical directions, totaling 1089 locations on the entire top surface of the quadruple cavity. For the resonance at 1514.715 nm (mode I), the field pattern is obtained by plotting the change of transmittance *∆T* as a function of position (*x*, *y*). As depicted in Fig. 3d–I, the field pattern shows a clear four-bounce diamond shape. Compared with the numerical results (Fig. 3e–I) and the Husimi map (Fig. 3f–I), we can confirm that the resonances in group-I are confined within the period-4 stable islands in sin *χ* ~ [0.6, 0.8]. By changing the probe wavelength to 1517.523 nm (mode II), 1533.596 nm (mode III), 1537.78 nm (mode IV), the corresponding field patterns have also been mapped and shown in Fig. 3d–II to Fig. 3d–IV. By comparing the experimental observations and their autocorrelation (see Supplementary Section 7) with the field patterns and Husimi maps, we can identify them as a stable six-bounce mode (Fig. 3e–II and Fig. 3f–II), a scar mode on the unstable six-bound orbit (Fig. 3e–III and Fig. 3f–III), and a scar mode on the unstable rectangular orbit (Fig. 3e–IV and Fig. 3f–IV). The analytical FSRs of these mode groups are shown in Fig. 3c, and their deviations from the experimental data reiterate the limitation of judging modes solely by their FSRs.

### Experimental confirmation of CAT

**Fig. 4:**
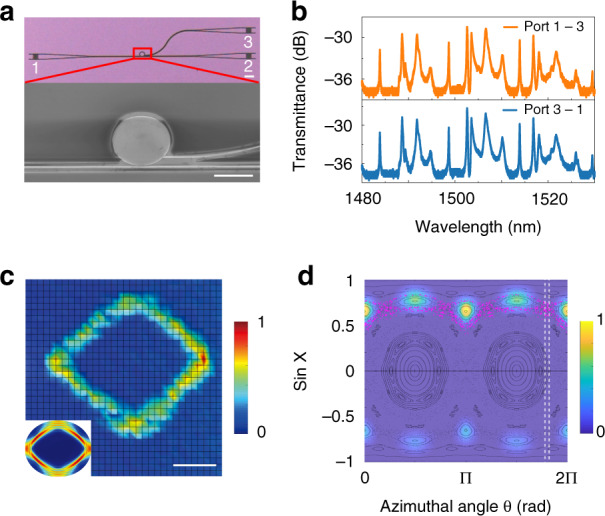
Direct observation of CAT. **a** Microscopic and top-view SEM images of the silicon microdisk with a channeling waveguide of width 445 nm. The scale bar is 10 µm. **b** Top: Transmission spectrum from the bus waveguide (port-1) to the channeling waveguide (port-3). Bottom: the same but from port-3 to port-1. **c** Correspondence of experimentally recorded field pattern and theoretically calculated mode pattern at *λ* = 1498.66 nm. The scale bar is 4 µm. **d** The Husimi map of the resonance at *λ* = 1498 nm. The ray dynamics are overlaid for a direct comparison, with the pink dots showing rays tunneled from the period-4 islands to the chaotic sea. The vertical box of white dashed line represents the leaky channel of the channeling waveguide

The ability to distinguish different resonances with similar FSRs demonstrated above is essential in the study of the CAT process. Figure 4a shows the microscope and top-view SEM images of a quadruple cavity of the same design, except for the addition of a channeling waveguide^18^ connected at *θ* = −36.3°. Light in many resonances can escape from this channeling waveguide (see the top panel in Fig. 4b), and the transmission peaks collected in this waveguide can also be separated into several groups with nearly identical FSRs. It is important to note that the majority of them are due to direct transport, which takes place when the channeling waveguide overlaps with the phase space structure of a mode in the PSOS, including modes II–IV studied in Fig. 3. Therefore, it is impossible to single out transmission peaks associated with CAT just based on the FSR.

Fortunately, the diamond modes are confined in the period-4 islands, which are clearly separated from the waveguide in the PSOS (see vertical box of white dashed lines in Fig. 4d). As a result, light in these modes can only escape via CAT and then be collected by the channeling waveguide at port-3. Therefore, the existence of CAT can be verified by observing a transmission peak in the channeling waveguide at the wavelength of a diamond mode determined by our method. One such confirmed CAT-induced transmission peak in Fig. 4b is at 1498.66 nm, and its clear diamond shape is shown in Fig. 4c (see transmission spectrum along the bus waveguide in Supplementary Section 9). This is the first time that a complete process including the evanescent coupling to modes in stable islands, the CAT, and the collection via channeling waveguide has been experimentally confirmed.

### The time-reversal process of CAT

The quadrupole microdisk in Fig. 4 is a time-independent system, described by a symmetric electric permittivity tensor (*ε*) and a symmetric magnetic permeability tensor (*µ*). In principle, this system should be constrained by the Lorentz reciprocity theorem^45–47^. Once the chaos is taken into account, the situation becomes totally different. The presence of chaos introduces inherent one-way stable structures in phase space. While individual trajectories are time-reversal, the averages in an experimental measurement invariably lead to (chaotic) attractor in the forward time direction, and the (chaotic) repeller in the reversed time direction^3,48^. Consequently, the CAT in optical microcavities is tend to be considered as an irreversible process^49^.

With the above technique, we are able to experimentally explore the time-reversal process of CAT for the first time. In this experiment, the experimental setup and the device are the same as the CAT experiment except that the probe light is sent in port-3 and the transmission spectrum is detected in port-1. The experimental results are plotted in the bottom panel of Fig. 4b. We observe again the transmission peak at 1498.66 nm with the same diamond mode. The backward transmittance is almost the same as the forward one. In fact, the entire transmission spectrum is very close to before (top panel of Fig. 4b) within measurement precision, providing strong experimental evidence of optical reciprocity^45,46^ involving CAT.

It is obvious that our experimental observation is in contradiction to the conventional belief of nonreciprocity in a chaotic system. This difference comes from our design. As depicted in Fig. 4d, the channeling waveguide introduces a new leaky channel marked by white dashed lines. The forward transmission is determined by the overlapping between chaotic layer surrounding the stable islands (pink dots) and the vertical box, which is far above the critical line and happens earlier than the refractive transmission^18,50^. Because port-3 is a single-mode waveguide, it can only collect a tiny fraction of the states with incident angles almost parallel to the waveguide. Such a small angular divergence is not sufficient to cause any observable breakdown of reciprocity in chaotic cavities.

## Discussion

In summary, we have demonstrated a powerful approach that reveals the internal field patterns of optical modes with drastically different dynamics, covering phase space structures such as unbroken KAM curves, stable periodic orbits, and the chaotic sea. This technique has been applied to confirm the CAT process with unprecedented assurance. Meanwhile, in contrast to conventional belief of chaotic system, the time-reversal process of CAT has also been verified for the first time. We also note that the main setup is similar to laser direct writing. Therefore, the in-plane laser spot size can be further improved to around 200 nm by optimizing the system with two-photon absorption and pulse duration (see Supplementary Section 6). The same technique can also be extended to active devices^51,52^ as well. We expect this research to offer new possibilities in the understanding of physical processes in optical microcavities and maximize the light–matter interactions within^53,54^.

## Materials and methods

### Numerical simulation

The resonances within the microdisks are calculated using the finite element method (COMSOL Multiphysics 5.4). We use a two-dimensional model with an effective index, and an outgoing boundary condition is implemented by perfectly matched layers applied outside the cavity. The eigenfrequencies (*ω*) of the resonances within the microdisks are then calculated with the photonics module, and their *Q* factors are derived using $Q = {\mathrm{Re}} \,(\omega )/\left| {2\,{\mathrm{Im}} \left( \omega \right)} \right|$. The wavefunctions on the cavity boundaries are also extracted to produce the Husimi map, obtained by projecting the wavefunctions to the phase space via the Husimi Functions.

### Device fabrication

The devices are fabricated on a SOI wafer with 220 nm top silicon device layer on 3 μm buried oxide. Then 340 nm photo resist ZEP 520 A is spin-coated onto the wafer as the soft mask. The silicon microdisks are patterned by the electron beam lithography (Raith E-Line) with 30 kV, then the sample was fully etched by using reactive ion etcher (Oxford, RIE80) (see details in Supplementary Section 1).

### Optical measurements

The incident light as a probe laser in the single-mode fiber is slightly tuned to the shorter side of the resonant wavelength and coupled into microdisk. Then the 420 nm nanosecond pump laser is focused on sample top surface through a 50× objective lens (NA = 0.42), which is fixed on an electric three-dimensional translation stage. The focal spot is about 610 nm, which is diffraction limited (see details in Supplementary Section 1).

## Supplementary information

Supplementary information for Direct Observation of Chaotic Resonances in Optical Microcavities

## Data Availability

All data needed to evaluate the conclusions in the paper are present in the paper and/or the Supplementary Materials. Additional data related to this paper may be requested from the authors.
