# Supplementary material for: Direct observation of chaotic resonances in optical microcavities
Source: Light Sci Appl. 2021 Jun 30;10:135. doi: 10.1038/s41377-021-00578-7 (PMC8241958; doi:10.1038/s41377-021-00578-7)
Supplement: Supplementary file 1 — Supplementary information for Direct Observation of Chaotic Resonances in Optical Microcavities [file 41377_2021_578_MOESM1_ESM.docx]

**Supplementary information for:**

**Direct Observation of Chaotic Resonances in Optical Microcavities**

Shuai Wang^1,†^, Shuai Liu^1,†^, Yilin Liu^1^, Shumin Xiao^1,2,3,¶^, Zi Wang^1^, Yubin Fan^1^, Jiecai Han^2^, Li Ge^4,5,*^, Qinghai Song^1,3,#^

^1^ Ministry of Industry and Information Technology Key Lab of Micro-Nano Optoelectronic Information System, Harbin Institute of Technology (Shenzhen), Shenzhen 518055, China.

^2^ National Key Laboratory of Science and Technology on Advanced Composites in Special Environments, Harbin Institute of Technology, Harbin 150080, China.

^3^ Collaborative Innovation Center of Extreme Optics, Shanxi University, Taiyuan 030006, China.

^4^ Department of Physics and Astronomy, College of Staten Island, CUNY, Staten Island, NY 10314, USA.

^5^ The Graduate Center, CUNY, New York, NY 10016, USA.

^†^ These authors contribute equally to this research.

Correspondence: ¶[shumin.xiao@hit.edu.cn](mailto:shumin.xiao@hit.edu.cn), *[li.ge@csi.cuny.edu](mailto:li.ge@csi.cuny.edu), #[qinghai.song@hit.edu.cn](mailto:qinghai.song@hit.edu.cn)

In the main text, we have studied the direct mapping of the resonances within circular and quadruple microdisks. With the assistance of an external perturbation introduced by a pump laser, we have shown that resonances with similar free spectral ranges in microdisks can be separated via their spatial field distributions. With this technique, we have clearly separated the regular mode confined within stable islands from their chaotic counterparts. As a result, the complete process of chaos assisted tunneling can be experimentally verified. In the supplemental information, we provide more experimental details and further elucidate the method to support the results presented in the main text.

**Section-1: The design and characterization of Si microdisks**

**1.1 Design of Si grating coupler**

The grating coupler is designed with a finite-element-time-domain (FDTD) method. In order to achieve a high *Q* factor for the microcavity, we choose the full etch process. Figure S1 shows the optimized nanostructures and the corresponding coupling efficiency. Near the designed central wavelength of 1510 nm, more than 30% of the incident laser can be coupled into the Si waveguide.

**1.2 Fabrication of Si microdisks**

The Si microdisks are fabricated with a combined process of electron beam lithography and reactive ion etching. The sample preparation procedures are summarized in Fig. S2. A 220 nm silicon on insulator (SOI) wafer is first cleaned with acetone, methanol, and isopropanol. Then 340 nm ZEP 520A is spin-coated onto the SOI wafer and baked at 180 ^o^C for 30 minutes. The microdisks, waveguides, and grating couplers are defined within the electron beam writer (Raith E-line) with 30 kV acceleration voltage. The patterns appear after developed in N50 for 60 s and MIBK for 20 s at room temperature.

The sample is dried with nitrogen gas and then placed into the reactive ion etcher (Oxford, RIE80). The etching gases are SF_6_ and CHF_3_ of 5 sccm and 50 sccm, respectively. The vacuum and the temperature are kept at 10^-5^ torr and 20 ^o^C. After 13 min of etching, the electron beam resist ZEP520A is removed with acetone and only the Si micro- & nano-structures are left. The sizes of circular and deformed microdisks have been described in the main text. Figure S3 shows the top-view scanning electron microscope (SEM) image of the grating coupler. Similar to the numerical design, the grating consists of nanogaps of different widths. A tapered waveguide is connected to the grating coupler to efficiently collect the coupled light.

**1.3 Optical setups for mapping resonances in Si microdisk**

In Fig. 1 of the main text, we have briefly introduced the optical setup and the working principle, and here we provide more details. Figure S4 shows the setup for the optical characterization. A tunable laser (Yenista TUNICS T100S-HP, 1440 nm to 1640 nm) is coupled to the waveguide via a single-mode fiber and the grating coupler. A reference grating coupler shows that the overall coupling in and coupling out losses are around -14 dB. The propagating light within the Si waveguide follows the fundamental waveguide mode and is coupled to the Si microdisks via the evanescent wave. By tuning the waveguide width and separation distance, the maximal coupling efficiency around 99% can be achieved, and transmission dips are formed at the resonant wavelengths when the light intensity from the grating out coupler is measured by a photoreceiver (New Focus, IR DC-125 MHz) and recorded with a digital oscilloscope (YOKOGAWA DLM2034, 2.5 GS s^-1^, 350 MHz).

Due to the limitation of the photoreceiver (125 MHz), we select a nanosecond laser (Continuum Surelite, 6 ns pulse duration, 10 Hz repetition rate) instead of a picosecond laser as the pump laser. The pump wavelength is fixed at 420 nm after considering the transmission window of our optical elements (visible range) and the stability of the shortest wavelength from our tunable nanosecond laser. When the local refractive index changed, the resonant wavelength of this mode shifts and thus the output power increases. One example is depicted in Fig. S5. When the pump laser is on and focused on one spatial intensity peak of the corresponding WGM, the increase of output power can be seen with the oscilloscope. This temporal peak also has a nanosecond duration and matches the pump laser well. Before and after the laser pulse, the intensity is on the noise level. This process clearly demonstrates that the field distribution of WGMs can be converted to wavelength shift, and finally to the intensity variation of the measured output power.

**Section-2: Influence of probe laser position**

Our probe wavelength (denoted by *λ_probe_* below) was slightly on the shorter wavelength side of each resonance, shifted by about one-tenth of the linewidth [see, for example, point 1 in Fig. S6b]. Actually, a *λ_probe_* further to the edge of the resonance can offer a more linear response in principle, with the underlying assumption that the shift ∆*λ*_0_ of the resonant wavelength is very small.

Take the ideal case of an isolated resonance at *λ*_0_ with linewidth Г*_λ_*, for example. The wavelength dependence of the transmission is given by

$T\left(\lambda;\lambda_{0}\right)=g\left[1-\frac{\left(\frac{\Gamma_{\lambda}}{2}\right)^{2}}{\left(\frac{\Gamma_{\lambda}}{2}\right)^{2}+\left(\lambda-\lambda_{0}\right)^{2}}\right]+\delta$ (S1)

where *g* is the overall coupling efficiency of the measurement and *δ* is the offset imposed by environmental and instrumental noises. Next, we choose *λ* = *λ_probe_*, and the sensitivity of the transmission measurement for a small change of *λ*_0_ can be characterized by the first-order derivative of the transmission, which we denote by $\partial_{0}T\left(\lambda_{probe};\lambda_{0}\right)\equiv\partial T\left(\lambda_{probe};\lambda_{0}\right)/\partial\lambda_{0}$. $\left|\partial_{0}T\left(\lambda_{probe};\lambda_{0}\right)\right|$reaches its maximum when $\partial_{0}^{2}T\left(\lambda_{probe};\lambda_{0}\right)\equiv\partial^{2}T\left(\lambda_{probe};\lambda_{0}\right)/\partial\lambda_{0}^{2}=0$, where the response is also most linear. Using the expressions

$\partial_{0}T\left(\lambda_{probe};\lambda_{0}\right)=\frac{\Gamma_{\lambda}^{2}\left(\lambda_{0}-\lambda_{probe}\right)}{2\left[\left(\frac{\Gamma_{\lambda}}{2}\right)^{2}+\left(\lambda_{0}-\lambda_{probe}\right)^{2}\right]^{2}}$ (S2)

$\partial_{0}^{2}T\left(\lambda_{probe};\lambda_{0}\right)=\frac{\Gamma_{\lambda}^{2}}{2}\frac{\left(\frac{\Gamma_{\lambda}}{2}\right)^{2}-3\left(\lambda_{0}-\lambda_{probe}\right)^{2}}{\left[\left(\frac{\Gamma_{\lambda}}{2}\right)^{2}+\left(\lambda_{0}-\lambda_{probe}\right)^{2}\right]^{2}}$ (S3)

we find that $\partial_{0}^{2}T\left(\lambda_{probe};\lambda_{0}\right)=0$ is reached at $\left|\lambda_{probe}-\lambda_{0}\right|=\Gamma_{\lambda}/2\sqrt{3}\sim0.29\Gamma_{\lambda}$ [red arrows in Fig. S6a]. This is where one wants probe wavelength to be (i.e., $\lambda_{probe}=\lambda_{0}\pm0.29\Gamma_{\lambda}$) if ∆*λ*_0_ is very small.

To facilitate the observation of the change to the transmission intensity, the shift of the resonant wavelength needs to be significant. Therefore, we choose the largest wavelength shift in our experiment to be around ∆*λ_0, max_* ~ 0.25Г*_λ_*, achieved by tuning the power of the pump laser. With such a setup, the small-signal analysis presented above requires revision. For this purpose, we then derive from Eq. (S1) the following expression for the shift of the resonant wavelength ∆*λ_0_* = *α*Г*_λ_* with *λ_probe_* at *λ*_0_ – *u*Г*_λ_*:

$\Delta T\left(\alpha\right)=T\left(\lambda_{probe};\lambda_{0}+\Delta\lambda_{0}\right)-T\left(\lambda_{probe};\lambda_{0}\right)=\frac{4\alpha g\left(2u+\alpha\right)}{\left(1+4u^{2}\right)\left[1+4\left(u+\alpha\right)^{2}\right]}$ (S4)

Using *u* = 0.11 (as in our experiment; solid line) and 0.29 (the optimum in the small-signal analysis; dashed line), we plot ∆*T* (*α*) in Fig. S7a. Both of them are almost linear, with the same residue *r*^2^ = 0.9978 from the least-square fitting accidentally. Therefore, we do not expect a noticeable enhancement by placing the probe wavelength further towards the edge of the resonance.

This expectation is shown explicitly in Fig. S7c and S7d. They plot ∆*T* (scaled by *g*) with *u* = 0.11 and 0.29 respectively, using the values of ∆*λ*_0_ simulated for the diamond mode shown in Fig. S7b. We find that both images capture the essential features of the diamond orbit, and they are arguably more recognizable than the actual mode pattern itself. Here the maximal wavelength shift is chosen to be 0.25Г*_λ_* as in our experiment, and we consider a redshift of the resonance for a reason to be explained later. Note that we consider here a standing-wave mode pattern instead of a traveling-wave one for simplicity.

**Section-3:** **Research on free carrier absorption and thermal effect**

In order to determine whether the signal we extract is caused by free carrier absorption (FCA) or thermal effect, we place the probe laser to the different positions of the resonance peak. The results are shown in Fig. S8 below.

When the probe wavelength is at point 2 in Fig. S6b, we observe an initial dip in the transmission before it rises to its peak (see Fig. S8b). The dip is too deep to be the result of the noise, and hence we attribute it to the FCA induced blueshift of the resonance (with a reduction of the refractive index inside the cavity). The subsequent peak in the transmission is then due to the thermal effect induced redshift of the resonance (with an increase of the refractive index). This explanation is further confirmed by placing the probe wavelength on the other side of the resonance (e.g., point 3 in Fig. S6b): now the initial fast blueshift due to FCA will lead to a peak of the transmission (Fig. S8c), opposite to that shown in Fig. S8b. Similarly, the following redshift of the resonance due to the thermal effect should cause a dip of the transmission, which is also observed in Fig. S8c.

In Fig. S8a, we believe that there is also an initial dip in the transmission, when the probe wavelength stays shorter than the blue-shifted resonant wavelength. The amplitude of the dip, however, is too small to be discerned from the noise. When the blueshift of the resonant wavelength (e.g., 0.25Г*_λ_*) is larger than 0.11Г*_λ_*, the probe wavelength is initially shorter and then longer and finally shorter again than the blue-shifted resonant wavelength, when the later comes back to its original value. This behavior should cause two dips in the transmission intensity, but their amplitudes are again on the order of the noise and cannot be discerned in the experimental data. In both cases, the subsequent peak of the transmission, which is caused by the thermal effect, is used to map the mode patterns inside the cavity.

**Section-4: Phase space structures of the quadruple cavity**

In the main text, we have discussed the phase space structures of an optical microcavity. Here we take the quadruple cavity *ρ*(*θ*) = *R* (1 + *ε* cos2*θ*) as an example to illustrate it. When *ε* = 0, the quadruple cavity is actually a circular cavity (see Fig. S9a). By recording the polar position *θ* and sine of the incident angle *χ*, the Poincaré surface of section (PSOS) of the cavity is plotted in Fig. S9b. A series of straight lines can be seen, presenting the whispering gallery modes (WGMs) in circular microcavities. With a gradual increase of deformation parameter *ε*, the ray dynamics do not change to completely chaotic immediately. At a small *ε*, the straight lines change to unbroken Kolmogorov-Arnold-Moser (KAM) curves, representing quasi-WGMs in deformed microcavities. With the further increase of *ε*, chaotic regions first appear at small sin *χ* and the size of the chaotic sea increases with *ε*. When *ε* is above 0.11, the phase space structures become fully chaotic. This transition process has been thoroughly discussed in the literature.

Here we mainly focus on the case with *ε* = 0.08. The quadruple cavity shape is plotted in Fig. S9c. The corresponding phase space structures are shown in Fig. S9d. As mentioned in the main text, the phase space is mixed with regular states mostly at large sin *χ* and the dominant chaotic sea at smaller sin *χ*. For the intermediate range of sin *χ*, there are closed curves, which are known as stable islands and surrounded by the chaotic sea. The predominant ones above the critical angle are the period-4 stable islands, which correspond to the diamond modes studied in the main text and previous works. We note that the period-6 stable islands have only been studied in theoretical analysis or numerical calculations. In Fig. 3 of the main text, we have shown their field patterns experimentally for the first time.

**Section-5: Dependence of resonant wavelengths on the local refractive index change**

The basis of our proposed method is the conversion of field distributions to the wavelength shift under a fixed external perturbation of the refractive index. First of all, we numerically and analytically verify that the field pattern won’t be destroyed by the change of refractive index. We simulated a quadruple cavity of radius *R* = 4.6 μm and deformation *ε* = 0.08. We use *n* = 2.84 as the cavity index and a perturbed disk region of a 600 nm diameter. The change of refractive index is Δ*n* = -0.01. The results are shown in Fig. S10. It is clear that the period-4 mode near *λ* = 1524 nm does not show a strong modification in its pattern. As a result, the wavelength shift can reflect the mode pattern under the external perturbation.

In this simulation, we treat the microcavity as two-dimensional (2D) and focus on the transverse electric (TE) modes inside the cavity, defined by

(S5)

with a proper outgoing boundary condition. Here *ϕ_m_* (*x, y*) is the component of the *H* field in the *m*th mode perpendicular to the 2D plane, *n* is unperturbed cavity refractive index, *c* is the speed of light in vacuum, and *ω_m_* is the complex mode frequency. Outside the cavity, the refractive index is taken to be 1, and the escaped light satisfies the same equation but with *ω_m_* replaced by the real-valued frequency of the probe laser. {*ϕ_m_*} so defined are sometimes referred to as the constant-flux (CF) states, and they satisfy the following bi-orthogonal relation when the refractive index is a constant and the system is away from an exceptional point:

(S6)

Now with the local index perturbation Δ*n* (*x*, *y*; *x*_0_, *y*_0_) introduced by the pump laser centered at (*x*_0_, *y*_0_), a perturbed mode can be expanded in the original, unperturbed CF basis, i.e*.*,. Substitute it into

(S7)

and perform an integration inside the cavity with an additional factor of , we find

(S8)

using the biorthogonal relation (S6). In the absence of strong mode mixing, the numerator can be approximated using just the *m* = 0 term, leading to Eq. (1) in the main text.

We then numerically verify Eq. (1) in the main text here using a quadruple cavity of radius *R* = 4.6 μm and deformation *ε* = 0.08. We use *n* = 3.74 as the cavity group index and a perturbed disk region of radius *r* = 0.3 μm and ∆*n* = 0.013 (due to the thermal effect). By scanning along the short axis of the quadruple cavity, the simulated wavelength shift of a diamond mode at 1509 nm using COMSOL is plotted in Fig. S11a. It agrees very well with the result of Eq. (1). Note that we consider here the standing-wave mode pattern instead of the traveling-wave one for simplicity.

Next, we repeat the procedure by scanning the entire mode using a 33 by 33 grid, obtaining its simulated wavelength shift as a function of the spatial position. The calculation is performed with the Eq. (S1). Here the measured transmission curve of a diamond mode is fitted with the following parameters, i.e. *g* = 0.5%, *δ* = 3.5 × 10^-4^ and Г*_λ_* = 0.315 nm. The result is plotted as the dashed line in Fig. S11b. We then choose *λ_probe_* = *λ*_0_ – 0.11Г*_λ_* as in the experiment and utilize ∆*λ*_0_ already obtained from the COMSOL simulation to calculate following Eq. (S4) and Eq. (1) in the main text. The resulting ∆*T* (scaled by *g*) is plotted in Fig. S11c, from which the diamond orbit is clearly seen.

**Section-6: The limitation of resolution**

Because light in our silicon microdisk is mostly traveling waves along one direction, the modes are more or less uniform along the propagation direction. Their spatial features are then defined predominantly by the lateral sizes of the beam-like modes. For the sizes of microcavities and the wavelength range we explore, the typical lateral size of these modes is on the order of a few hundred nanometers to 1 μm. Therefore, the experiments in the main text do not require a high resolution to identify different types of resonances, such as WGMs with different radial numbers in a circular microdisk or quasi-WGMs and chaotic modes in a quadrupole microdisk. However, it is still interesting to discuss the limitation of spatial resolution and the possibility to further improve it. This can enable the visualization of all the sub-wavelength patterns.

The first limitation of the current setup is the spot size of pump laser. According to the Rayleigh formula, the diffraction limit size can be calculated by the following formula:

$D=\frac{0.61\lambda}{NA}$ (S9)

The spatial laser spot size is approximately equal to 610 nm with *λ* = 420 nm, NA = 0.42, which has been experimentally confirmed. The measurement result is shown in Fig. S12(a). Extracting the intensity curve along the diameter of the spot, we performed Gaussian fitting of the data. As depicted in the Fig. S12(b), the FWHM of the fitted curve is 599.6 nm, matching the theoretical calculation very well. The focal spot size can be improved. By applying shorter wavelength and larger NA objective lens, the spot size can be reduced to 100-200 nm. Due to the diffusion of thermal effect, the final resolution is also related to thermal diffusivity in silicon, which is mainly determined by temperature. To measure the temperature at the laser spot, we heat the sample to be tested and measure the wavelength shift of the diamond mode. The results are shown in Fig. S13a below. According to the fitting result in Fig. S13b, the wavelength shift coefficient of the diamond mode with temperature is 0.05 nm ℃^-1^. When the nanosecond laser is on, we collect the response signal and move the detection wavelength to keep the maximum transmission intensity of the two consistent. The result is shown in Fig. S14. At this time, it can be confirmed that the wavelength has red-shifted by 0.1 nm. Considering that the radii of the pump laser spot and the microcavity are about 300 nm and 7 μm respectively, the temperature at the laser spot can be calculated to be 1362 K, which is lower than the melting point of silicon 1687 K. Given the thermal diffusivity of silicon at this temperature (0.12 cm^2^ s^-1[1]^), we find the that the distance of thermal diffusion to be 618 nm in 100 nanoseconds. However, we should note that as heat dissipates, especially in the presence of efficient cooling, the effect of thermal diffusion on the feature size of local index perturbation (and the resolution in our approach) will be much weaker.

The second limitation of our setup is the response of the power meter. Our current photodetector is only 125 MHz, which is slow and can only be associated with the nanosecond laser. If a 50 GHz photodetector is applied, we can use the 20 ps pulse laser as the pump laser. In this case, the heating effect can be neglected and the wavelength shift will be dominated by the free carrier effect within the pump laser spot. In principle, the normal temperature diffusion time of carrier is relatively short and the final resolution can be kept at ~ 200-300 nm, 1/5-1/6 of the probe wavelength. This is good enough to resolve most of the fine structures.

In addition, the current repetition rate of nanosecond laser is 10 Hz, which strongly limits the scanning speed. For a typical Galvo Scanning Mirror, the scanning frequency is larger than 1 KHz. In this sense, using a high repetition rate laser such as Ti:Sapphire laser with 80 MHz, the scanning speed can be even faster. This is one of the intrinsic advantages of the demonstrated technique. It doesn’t need to contact the surface or reach the evanescent waves. It is not necessary to precisely control the vertical distance between the perturbation tip and microdisk. Thus, the scanning speed can be orders of magnitude faster than the scanning near field optical microscope (SNOM) and the scanning perturbation imaging technique in the microwave regime.

**Section-7: Autocorrelation field patterns**

Modes II and III shown in Fig. 3d of the main text are very similar by eye. However, this does not mean that they cannot be distinguished with high confidence once a quantitative comparison is performed. As an example, here we apply autocorrelation in image processing to enhance the geometric shapes of the corresponding ray orbits. This approach is suitable when the image has repeated features. Take the 1D signal $I(x)$ in Fig. S15 for example, which has two square “pulses”. Its autocorrelation function$C_{I}(x)$, proportional to the overlapping integral of the signal and its shifted copy, i.e.,

$C_{I}(x)=\frac{\int dx'I(x')I(x'+x)}{\int dx'\left|I(x')\right|^{2}}$ (S10)

is peaked at the $x=0$ as expected. However, a more important feature is that the two square “pulses” are now transformed to two triangular “pulses” highlighting their central positions (even though their values are doubled due to the autocorrelation function in this case).

The 1D example above can be thought as a 1D slice of the 2D images shown in Fig. 3d of the manuscript through the center of the cavity. Therefore, the center of the propagating beam will be enhanced after performing the autocorrelation in 2D, leading to a “purer” shape of the orbit as we show in Fig. S16: The autocorrelation pattern on the left is produced using the field pattern of mode II, and it shows clearly the stable six-bounce orbit supporting mode II. On the contrary, the autocorrelation pattern of mode III shown on the right is clearly different, and the unstable six-bounce orbit can be identified especially by inspecting the inner edge of the pattern.

**Section-8: Research on modes Q factors with field mapping technique**

By Lorentz fitting the resonances spectra, the FWHMs of the mode I to III measured in Figure 2b are 0.110 nm, 0.305 nm, 0.145 nm and the corresponding Q factors are 13759, 4950 and 10347, respectively. In addition to the circular microcavity, we also measured the linewidths and *Q* factors of the quadruple microcavity. The FWHMs of the mode I to IV measured in Fig. 3d are 0.246 nm, 0.093 nm, 0.102 nm, 0.235 nm and the corresponding *Q* factors are 6157, 16317, 15035 and 6543, respectively. The corresponding experimental results are plotted in Fig. S17 and S18. Obviously, the *Q* factors of these modes in our experiment are relatively low. This is because the size of the microcavity we measured is small. At the same time, the *Q* factor of deformed microcavity is lower than circular cavity of the same size. If measure a larger microcavity, we need to scan more positions, which will increase the measurement time and this will be related to the stability of the current system. However, this does not mean that the method is not suitable for the mode of large *Q* factor and large samples, for example, we can use a faster photodetector and Galvo scanning mirror to increase the speed instead.

**Section-9: The transmission spectra between ports 1 and 2**

In Fig. 4, the diamond modes and the other chaotic resonances have been experimentally determined to determine the CAT and inverse CAT process. The identification of different modes is also performed by mapping the field pattern. As a result, the transmission spectra between ports 1 and 2 have been measured in experiments. Some of the results are plotted in Fig. S19. Since the coupling between waveguide and microdisk is optimized for the diamond, these modes dominated the resonant dips in Fig. S19. The transmission spectra from port-1 to port-2 is very similar to port-2 to port-1. However, the relative intensity is slightly different. Such a slight difference reflects that the CATs of CW and CCW components in stable islands are slightly different too, caused by the asymmetrical scattering of the channeling waveguide.

A more obvious difference happens between these two transmission spectra and the transmission spectra in Fig. 4(b) of the main text, where more resonant modes can be observed in the same microdisk. This difference is also understandable. As stated in the main text, the chaotic system itself is nonreciprocal if the averages of experiments are considered. In order to convert the nonreciprocity back to reciprocity, we have to select a very narrow channeling waveguide and restrict the states in the chaotic layer. As a result, the transmission efficiency of CAT between port-1 and port-3 is strongly scarified.

**Section-10: The repeatability of field mapping technique**

For field mapping in microcavity, the good repeatability is an essential character. To demonstrate the repeatability of our technique, we select the mode IV in Fig. 3d and scan the same microcavity four times, the patterns are shown in Fig. S20. It can be clearly seen that these four images are all scar mode on the unstable rectangular orbit. Therefore, we can confirm that our field mapping technique based on thermal effect have very nice repeatability.

**References:**

[1] Hull, R. *Properties of crystalline silicon*. (IET, 1999).

**Figures:**


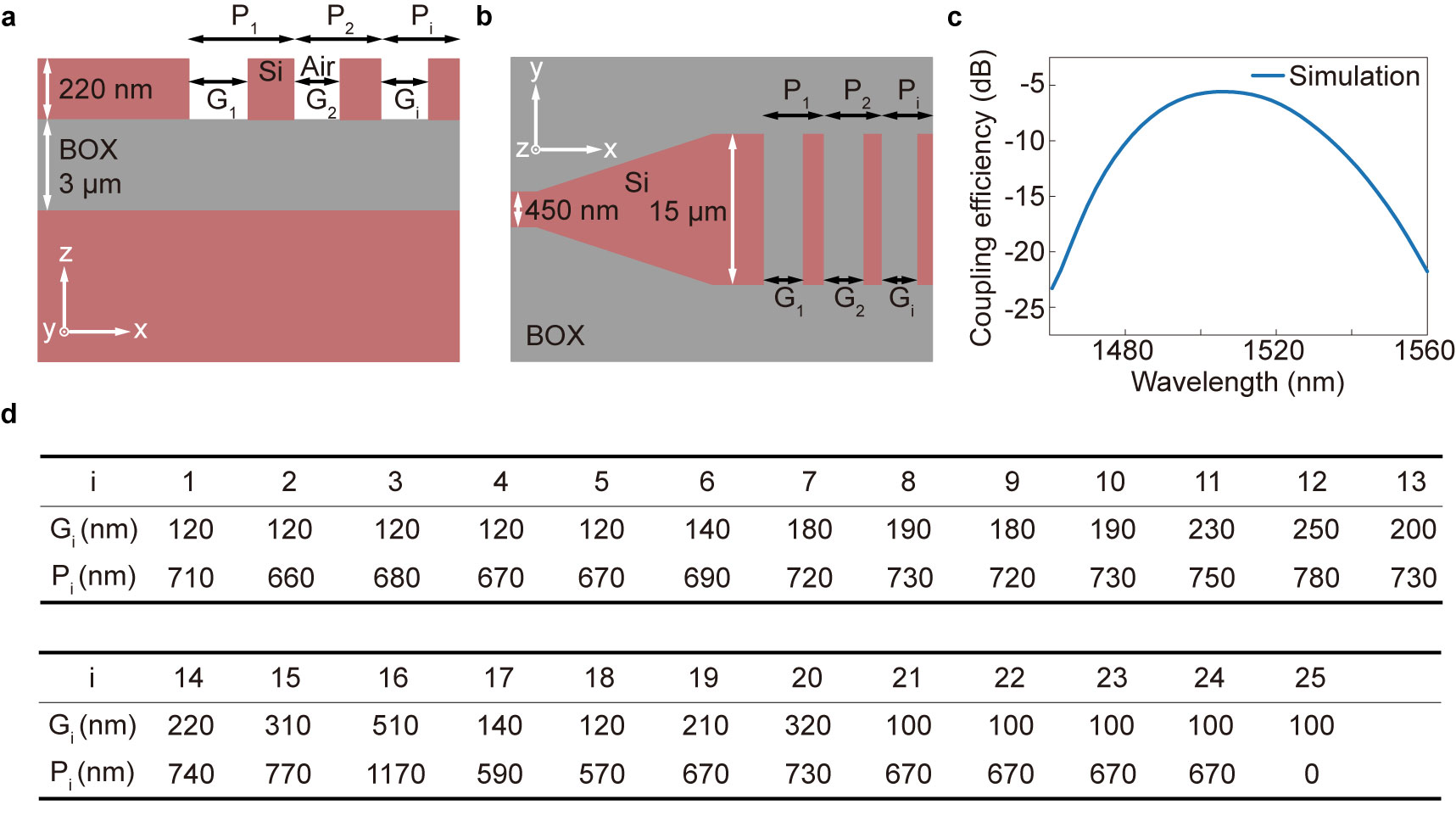


**Figure S1:** **Schematics of the fully etched silicon grating coupler. a.** Side view. **b.** Top view. **c.** Simulated coupling efficiency. **d.** Table showing the designing parameters of the grating coupler.


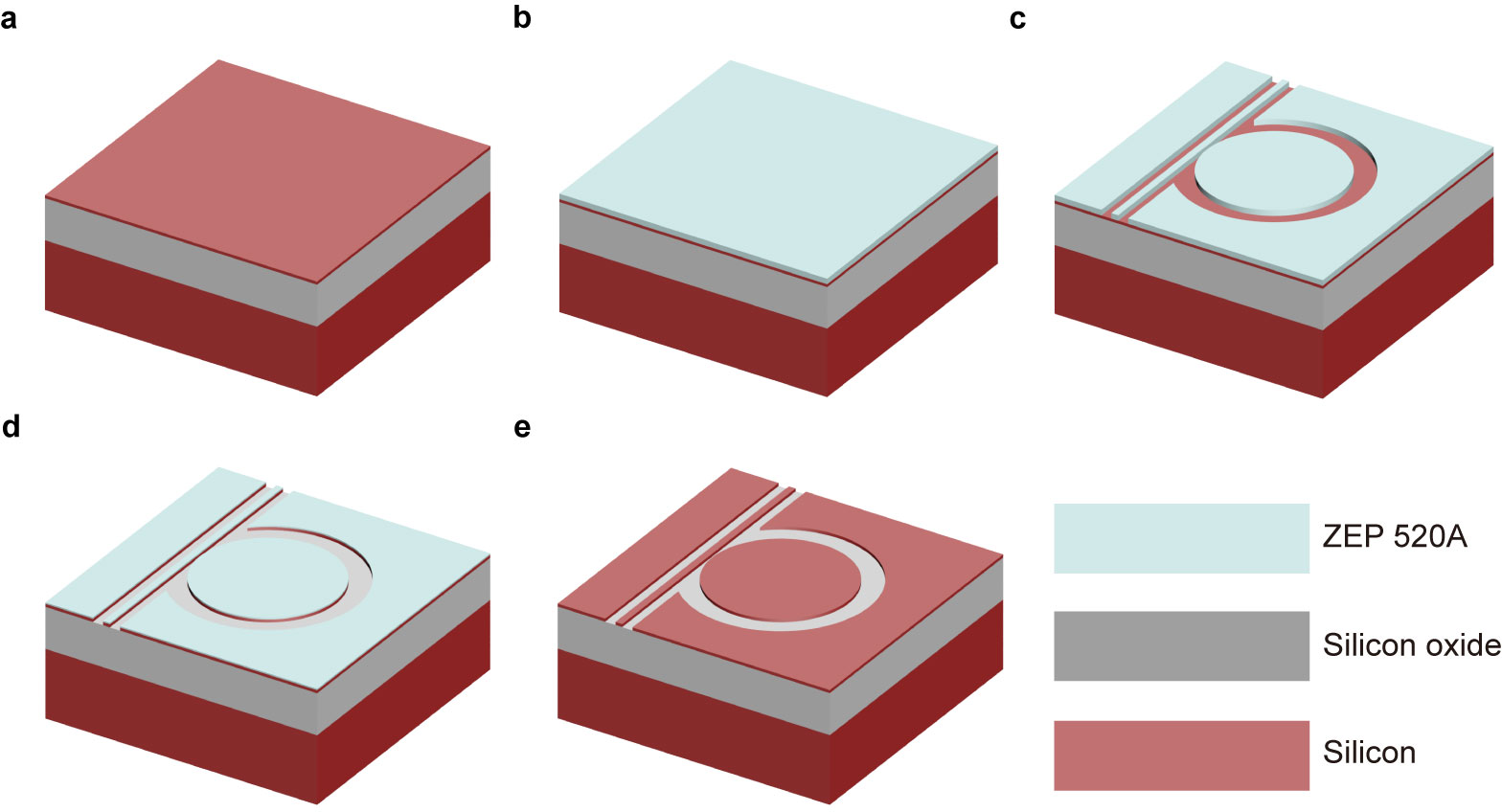


**Figure S2. Procedures for fabricating the Si microdisk. a.** Substrate cleaning. **b.** Spin coating. **c.** E-beam lithography. **d.** Dry etching. **e.** Photoresist removal.

**
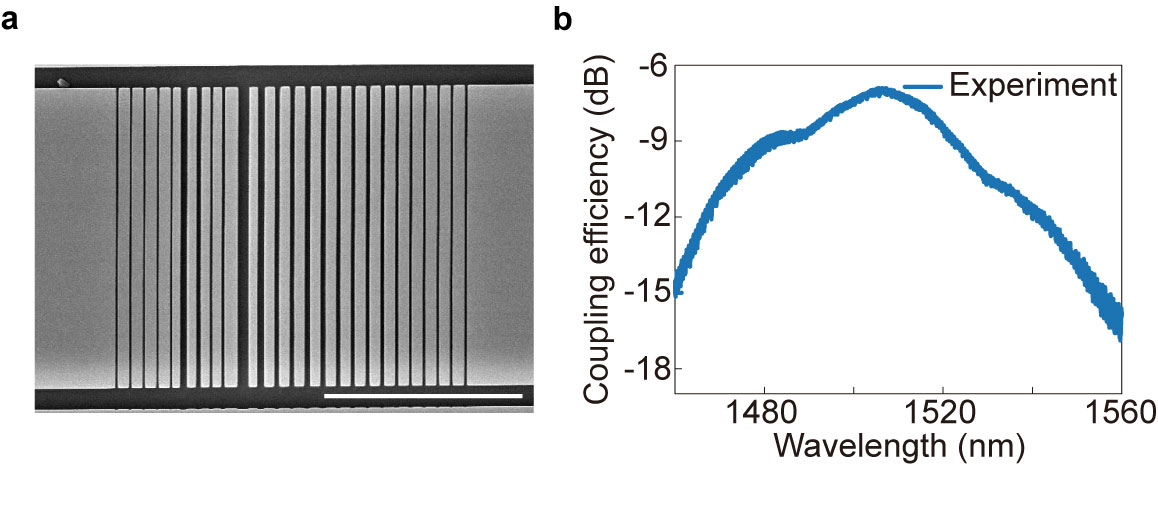
**

**Figure S3. Fabricated grating coupler. a.** Top view SEM of a non-uniform grating coupler. The scale bar is 10 µm. **b.** Measured coupling efficiency of a single grating coupler.


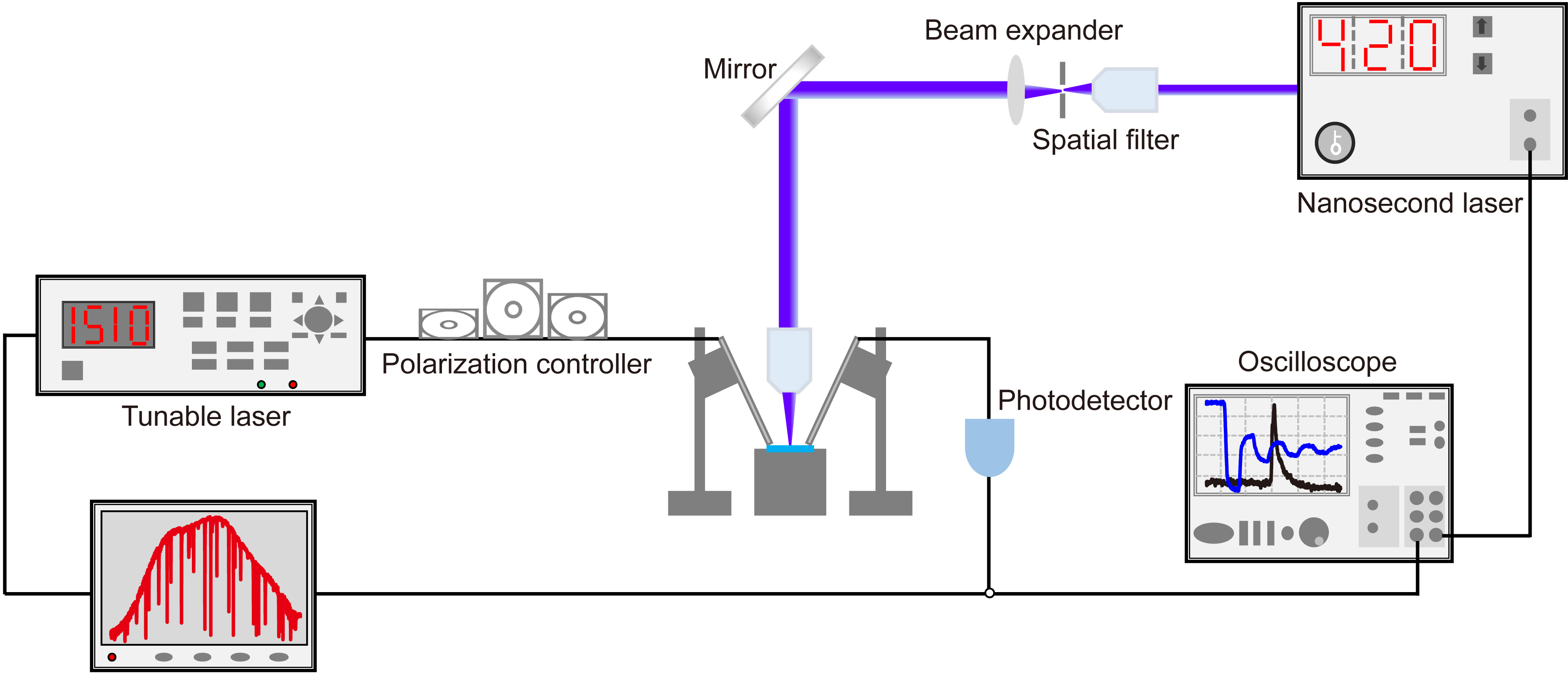


**Figure S4.** **Experimental setup for mapping­ the microcavity fields.**


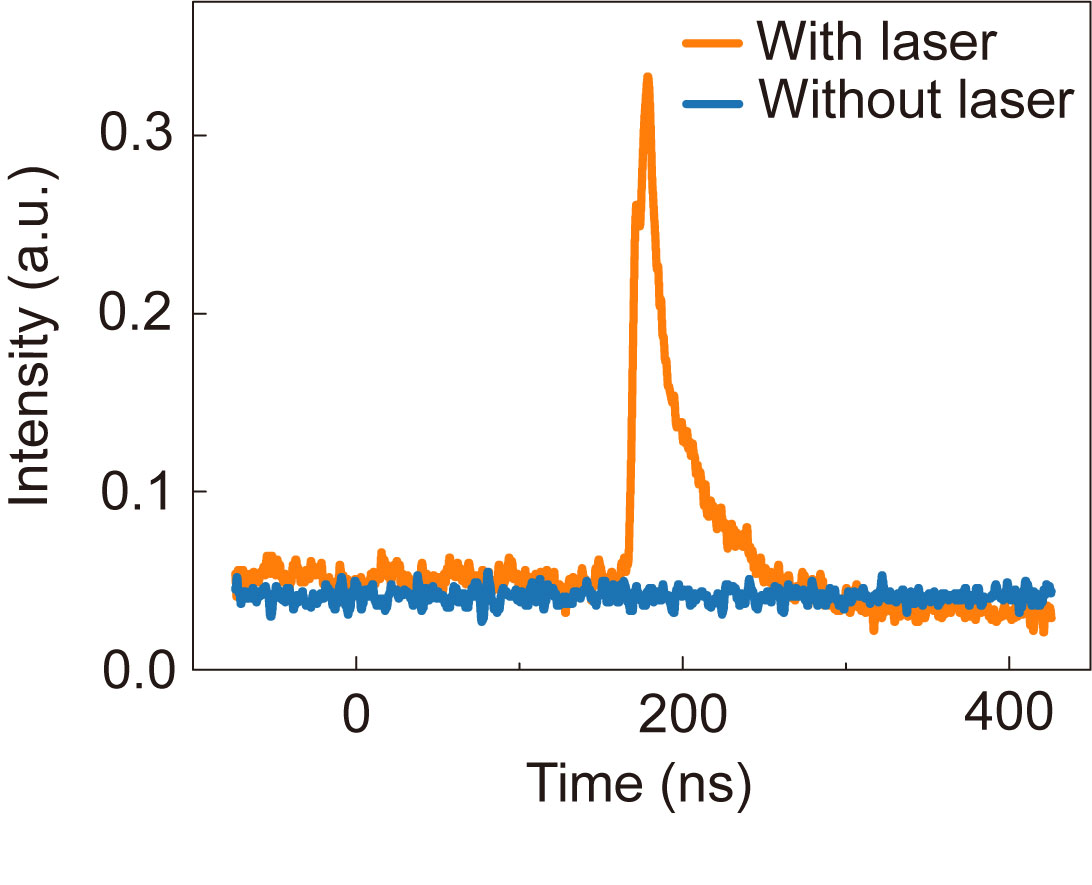


**Figure S5. Output power with and without the nanosecond laser pumping.**

**
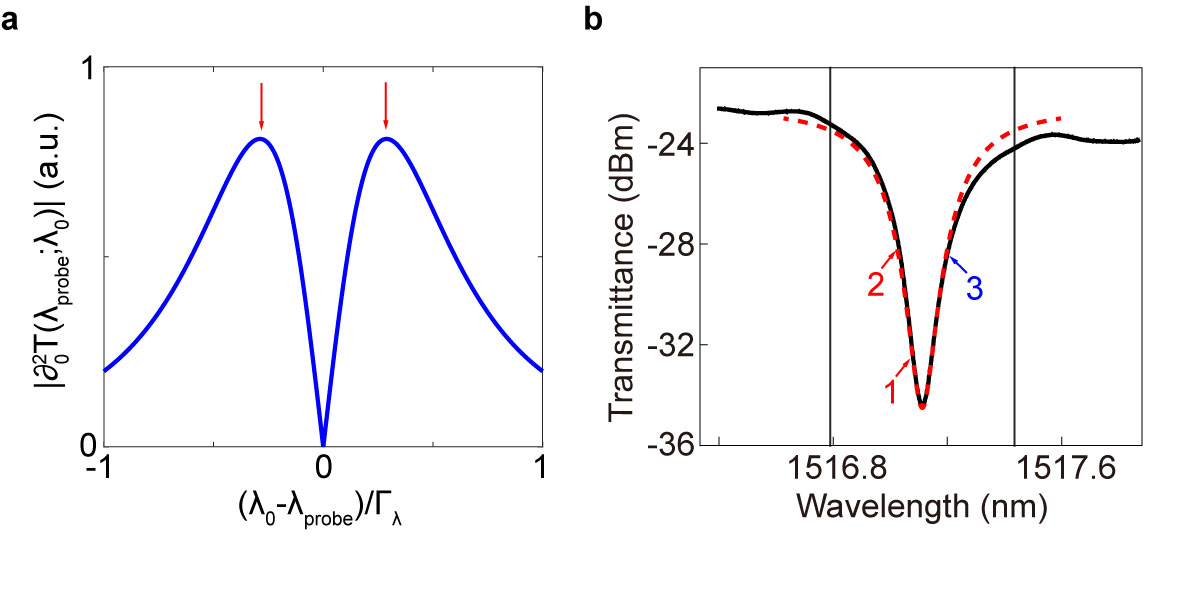
**

**Figure S6. Different probe positions. a.** Second-order derivation of the transmission *T* (*λ_probe_*; *λ*_0_) with respect to the resonant wavelength *λ*_0_ when the probe wavelength *λ_probe_* is fixed. **b.** Measured transmission near the resonance at *λ*_0_ = 1517.115 nm without the pump laser (black solid). Its fitting (red dashed) is obtained using *g* = 0.5%, *δ* = 3.5 × 10^-4^ and Г*_λ_* = 0.315 nm in Eq. (*S*1). The two vertical lines mark the edges of the resonance, at *λ* = *λ_0_* ± Г*_λ_*. Three values of the probe wavelength are indicated by the arrows, at *λ_probe_* = 1517.08, 1517.03, 1517.20 nm (points 1-3) that correspond to a difference of -0.11, -0.27, 0.27 times Г*_λ_* from the unshifted *λ*_0_.

**
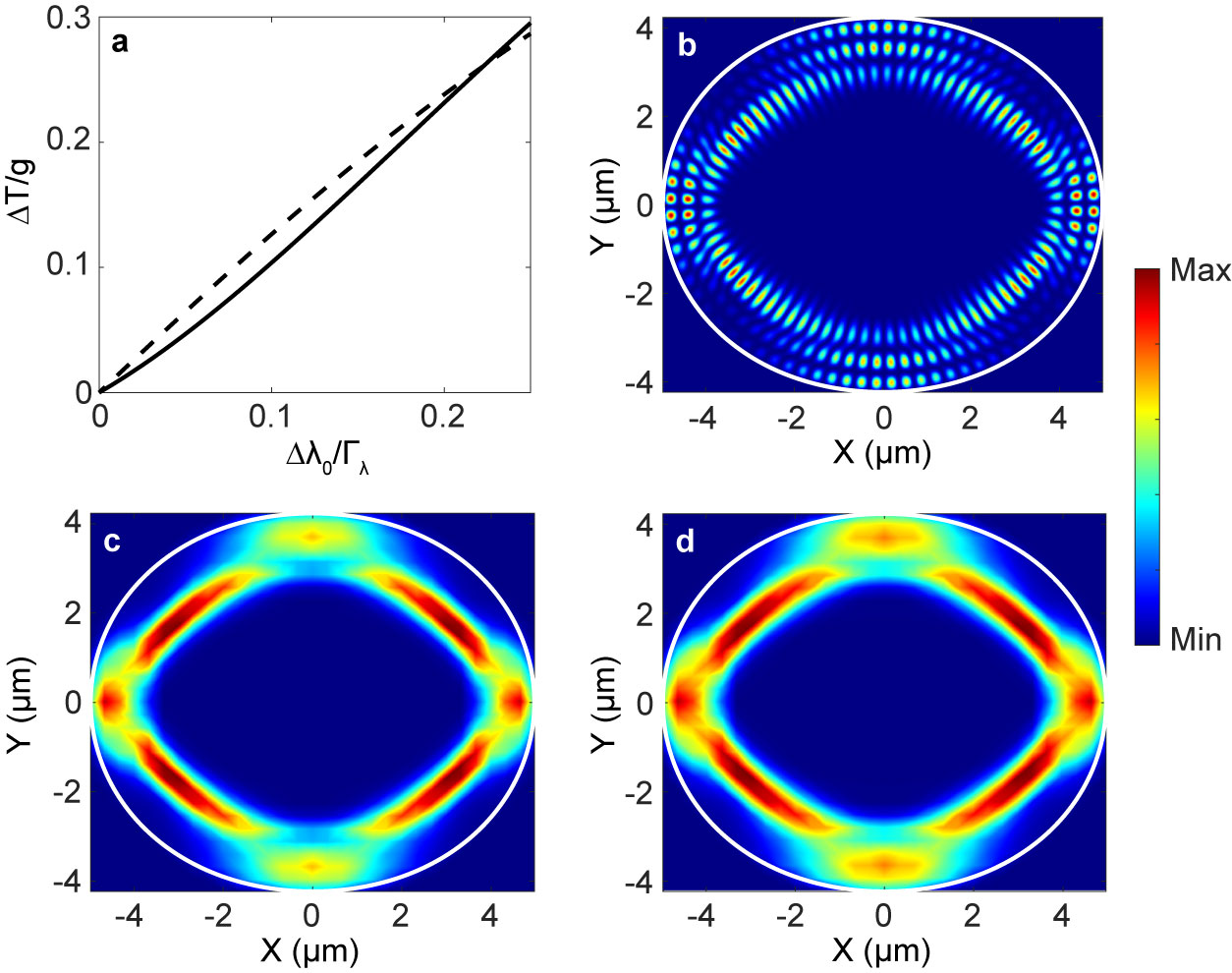
**

**Figure S7**. **Comparison of ∆*T* for two different values of the probe wavelength. a.** *λ_probe_* – *λ*_0_ = -0.11Г*_λ_* and 0.29Г*_λ_*. The maximum shift of the resonant wavelength is set to 0.25Г*_λ_*. **b.** The intensity pattern of a simulated diamond mode. **c, d.** Its images mapped by ∆*T*, with the probe placed at *λ*_0_ – 0.11Г*_λ_* in (a) and *λ*_0_ – 0.29Г*_λ_* in **c** respectively. The same colormap is used for these three configurations, with their respective minimum and maximum of ∆*T*.

**
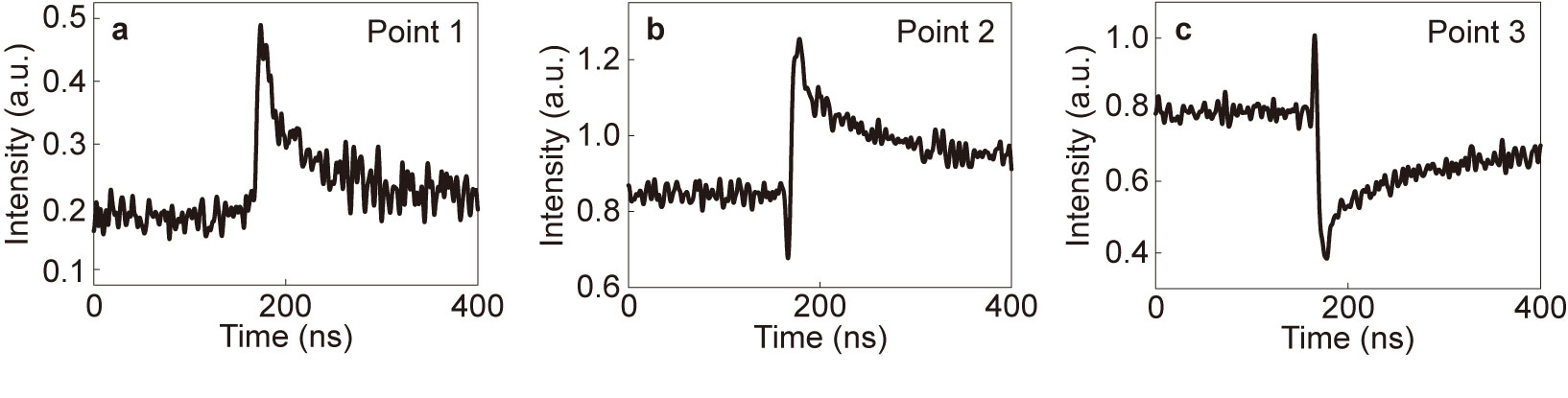
**

**Figure S8.** **Transient behavior of the transmission after the probe laser probed at different wavelengths marked in Fig. S6b.**


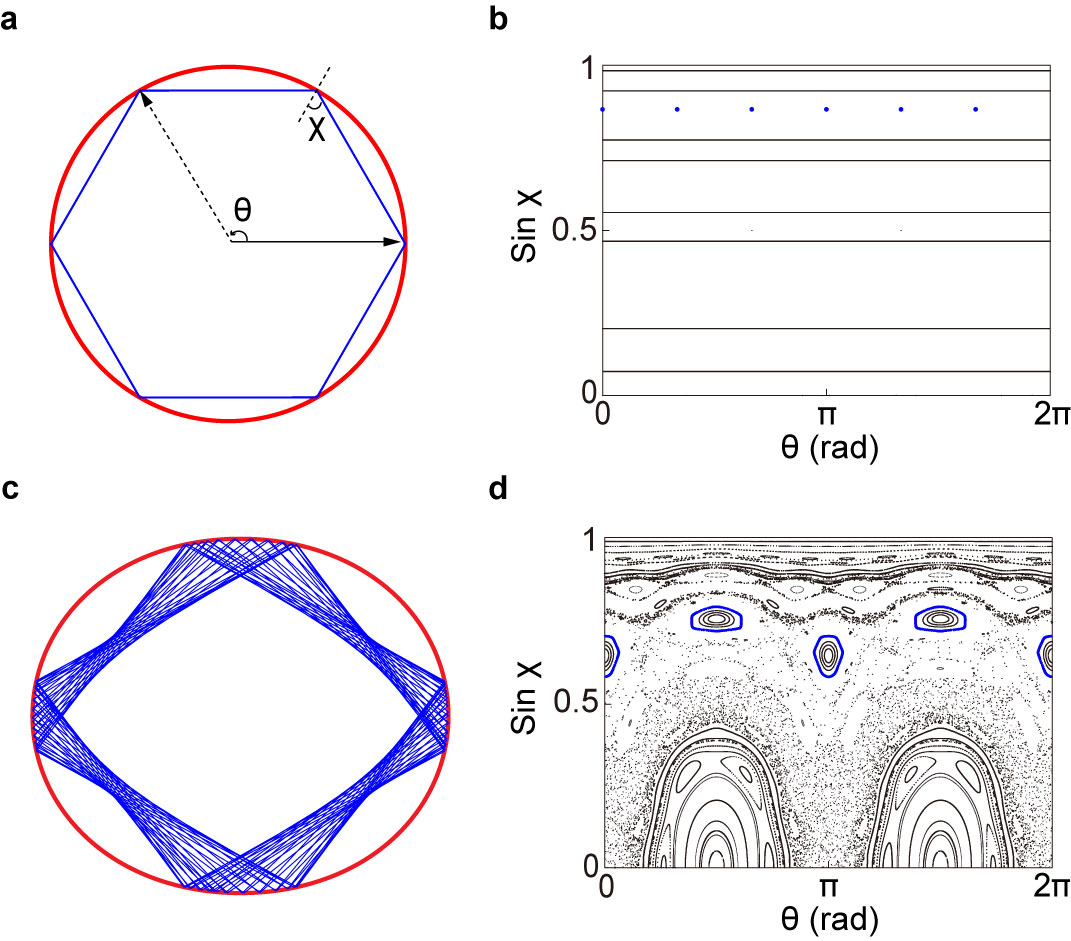


**Figure S9. Poincaré surface of section for the circular and quadruple cavities.** **a.** Schematic of the circular microdisk with the ray trajectory of a six-bounce mode. **b.** PSOS of the circular cavity. **c.** Schematic of the quadruple cavity with *ε* = 0.08, overlaid with the ray trajectory on the period-4 islands. **d.** Corresponding phase space structures in the PSOS.


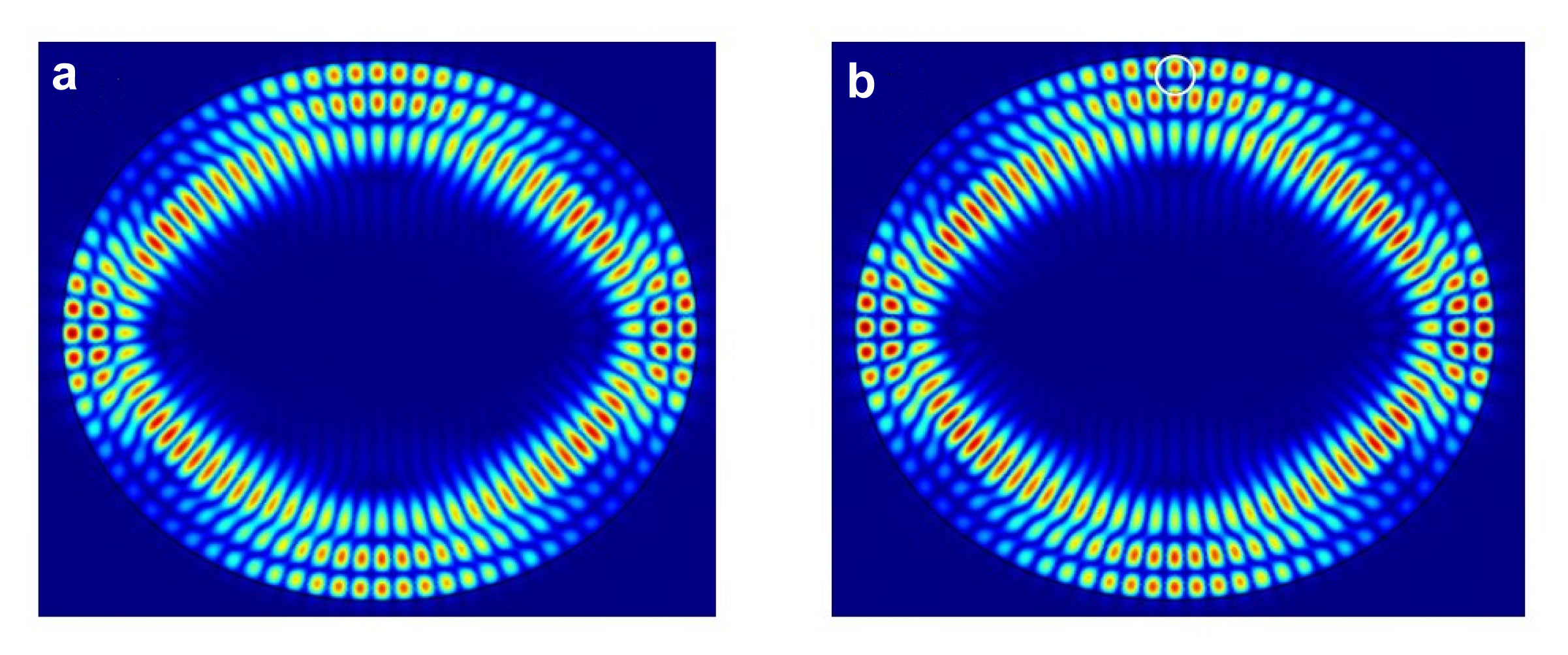


**Figure S10. The unperturbed (a) and perturbed (b) mode patterns of a period-4 mode.** The region of the local index perturbation is near the top of the cavity and marked by the white circle.


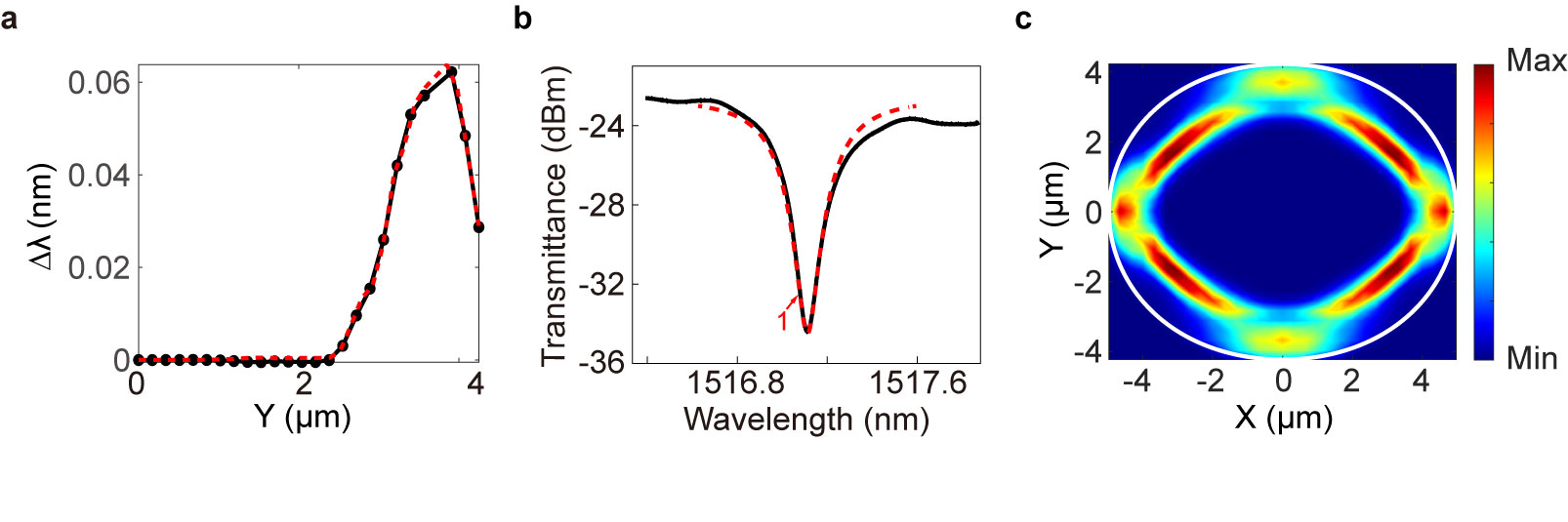


**Figure S11.** **Verification of frequency shift due to local index perturbation. a.** Black dots connected by the solid line show the numerical simulation from COMSOL. Red dashed line shows the analytical approximation given by Eq. (1) in the main text. **b.** Measured transmission near the diamond resonance at *λ*_0_ = 1517.115 nm without the pump laser (black solid). Its fitting (red dashed) is obtained using *g* = 0.5%, *δ* = 3.5 × 10^-4^ and Г*_λ_* = 0.315 nm in Eq. (*S*1). The probe wavelength at *λ_probe_* = 1517.08 nm is marked by the arrow, which is shorter than the resonance by 0.11Г*_λ_*. **c.** Mapped diamond mode at 1509 nm shown by ∆*T* using Eq. (1) in the main text and Eq. (S4).


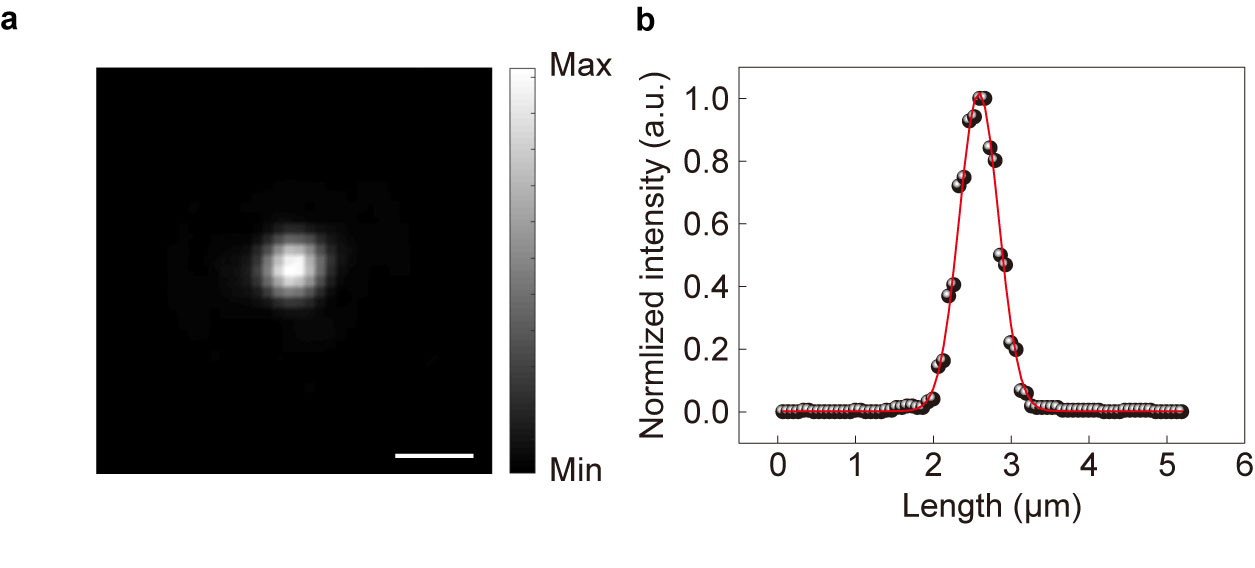


**Figure S12. Experimentally recoded focal spot.** **a.** Focal spot image collected by CCD, the scale bar is 1 μm. **b.** Gaussian fitting of intensity curve along the center of focal spot.


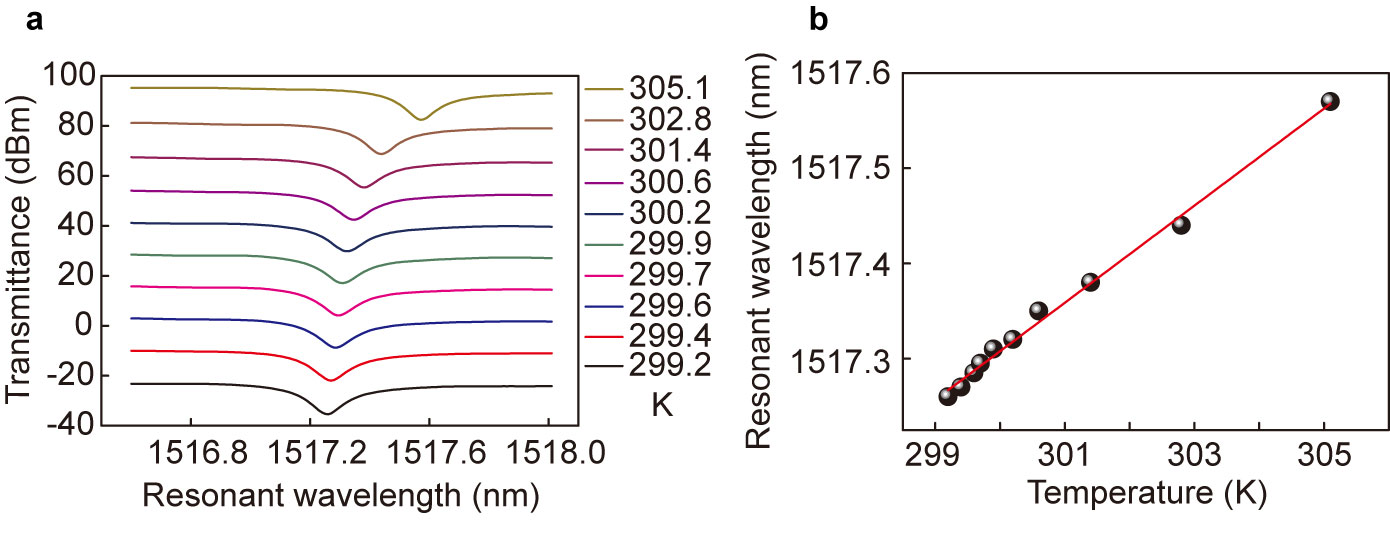


**Figure S13. The wavelength shift measurement of diamond mode at** **different temperatures. a.** Resonant mode transmittance spectra at different temperatures. **b.** The fitting result of wavelength shift coefficient with temperature.


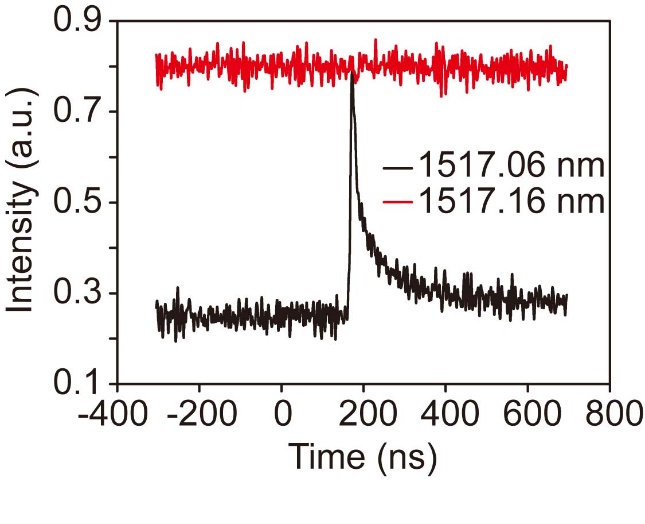


**Figure S14. The measurement result of the resonance wavelength shift by 0.1 nm.**

**
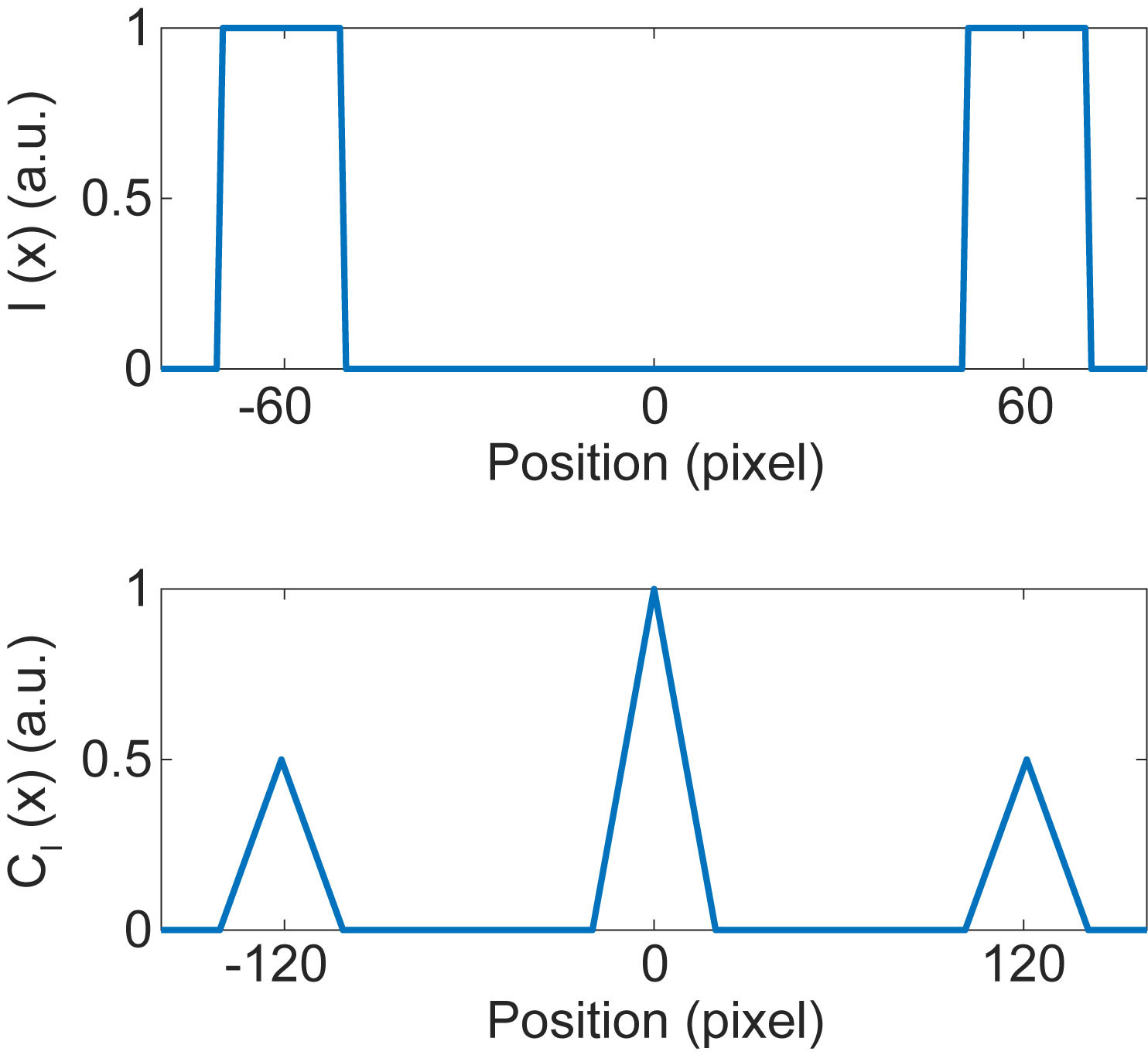
**

**Figure S15. Effect of autocorrelation for repeated patterns.**

**
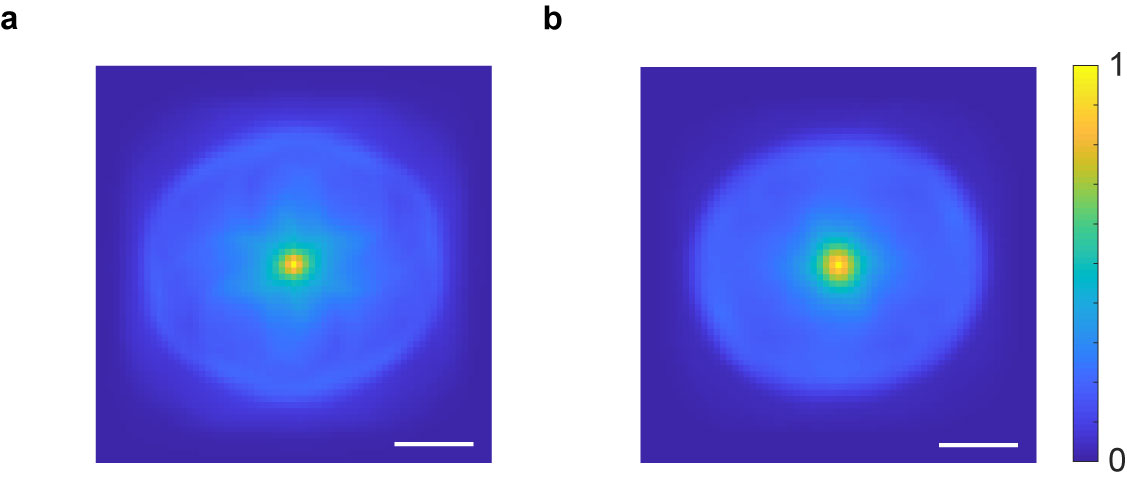
**

**Figure S16. The autocorrection figures of experimentally observed Mode-II (a) and Mode-III (b) in main text Figure 4, the scale bar is 4 μm.**


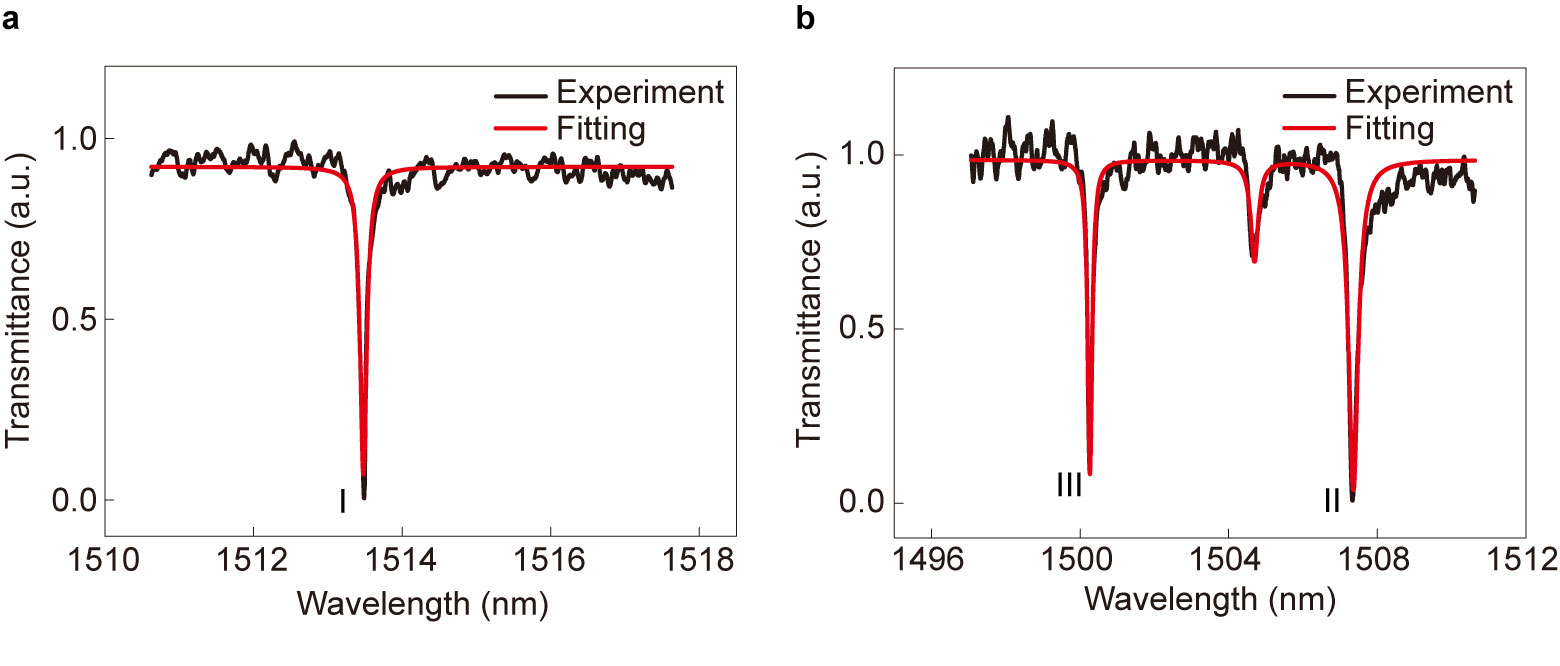


**Figure S17. The normalized linear spectrum of mode I to** **III shown in main text Figure 2b.** The red lines are the fitting results to accurately obtain the *Q* factors.


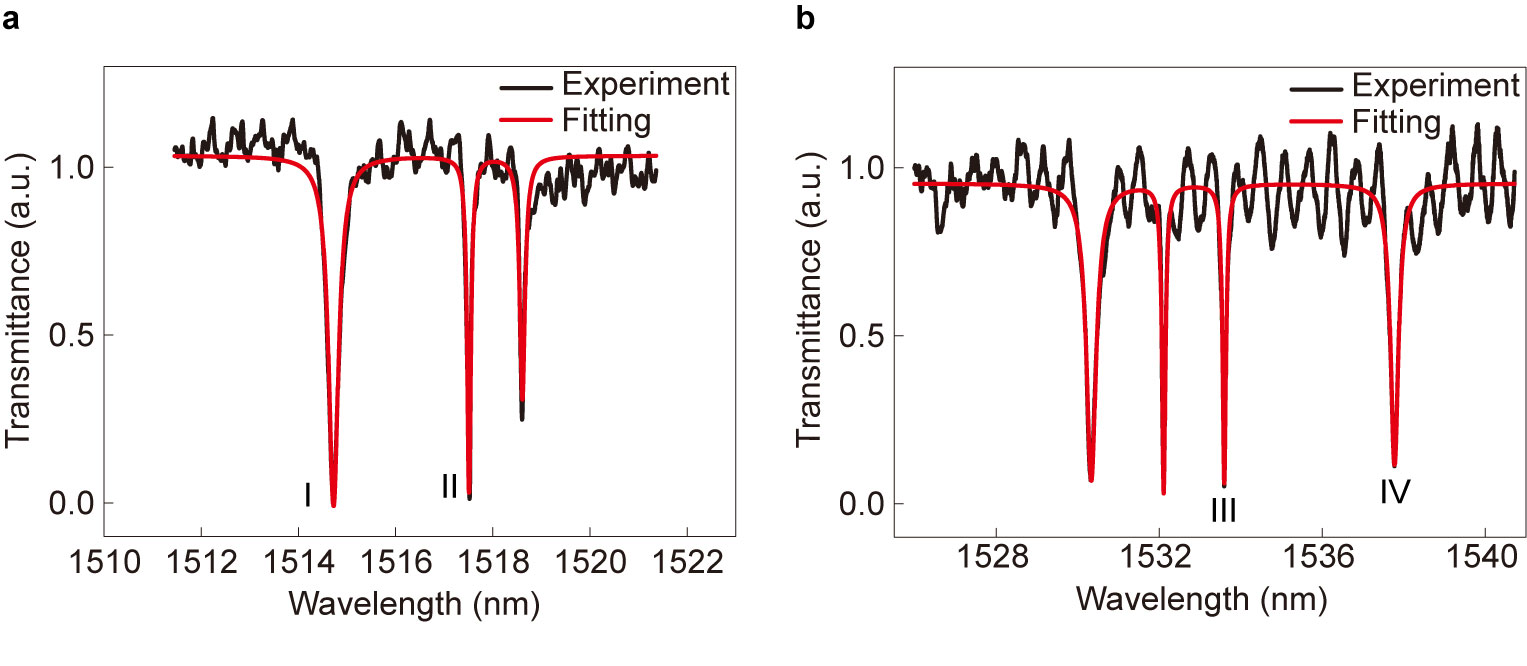


**Figure S18. The normalized linear spectrum of mode I to IV shown in main text Figure 3b.** The red lines are the fitting results to accurately obtain the *Q* factors.


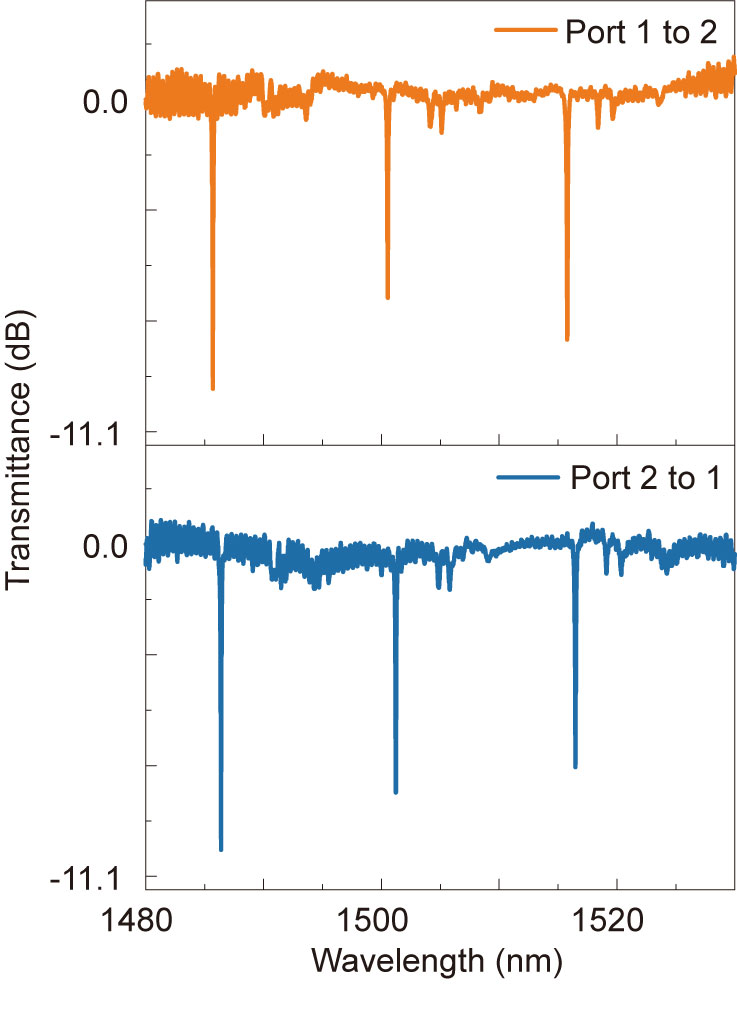


**Figure S19. The transmission along the bus waveguide.** Top: Transmission spectrum from the left bus waveguide (port 1) to the right (port 2). Bottom: the reversed transmission spectrum of the top one.


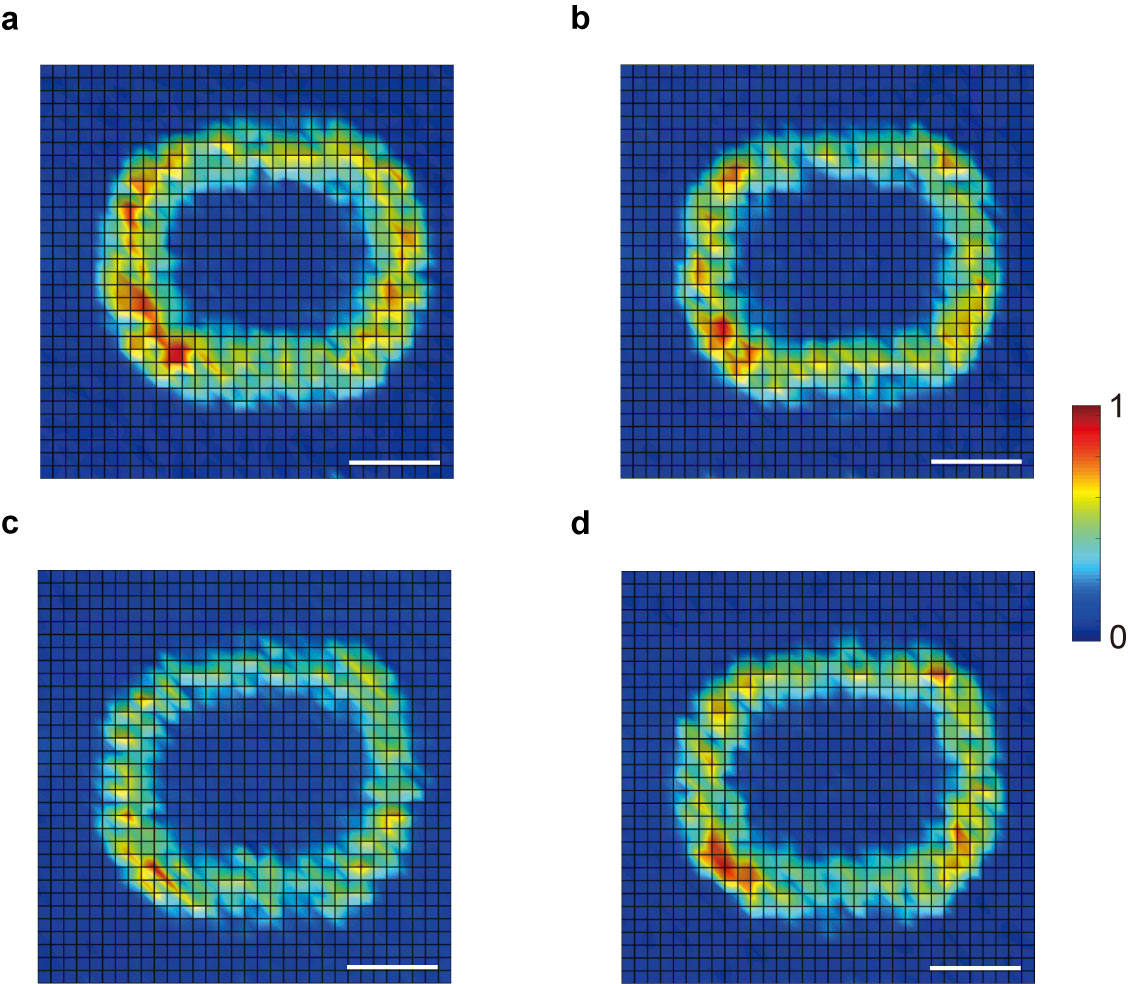


**Figure S20. Experimentally mapped field distributions of mode IV in main text Figure 3d for four times. All the scale bars are 4 µm.**
